# What the Spectrum of Microglial Functions Can Teach us About Fetal Alcohol Spectrum Disorder

**DOI:** 10.3389/fnsyn.2017.00011

**Published:** 2017-06-19

**Authors:** Elissa L. Wong, Rianne D. Stowell, Ania K. Majewska

**Affiliations:** ^1^Department of Environmental Medicine, University of Rochester Medical CenterRochester, NY, United States; ^2^Department of Neuroscience, University of Rochester Medical CenterRochester, NY, United States

**Keywords:** microglia, ethanol, neurodevelopment, plasticity, neuroimmune, synapse

## Abstract

Alcohol exposure during gestation can lead to severe defects in brain development and lifelong physical, behavioral and learning deficits that are classified under the umbrella term fetal alcohol spectrum disorder (FASD). Sadly, FASD is diagnosed at an alarmingly high rate, affecting 2%–5% of live births in the United States, making it the most common non-heritable cause of mental disability. Currently, no standard therapies exist that are effective at battling FASD symptoms, highlighting a pressing need to better understand the underlying mechanisms by which alcohol affects the developing brain. While it is clear that sensory and cognitive deficits are driven by inappropriate development and remodeling of the neural circuits that mediate these processes, alcohol’s actions acutely and long-term on the brain milieu are diverse and complex. Microglia, the brain’s immune cells, have been thought to be a target for alcohol during development because of their exquisite ability to rapidly detect and respond to perturbations affecting the brain. Additionally, our view of these immune cells is rapidly changing, and recent studies have revealed a myriad of microglial physiological functions critical for normal brain development and long-term function. A clear and complete understanding of how microglial roles on this end of the spectrum may be altered in FASD is currently lacking. Such information could provide important insights toward novel therapeutic targets for FASD treatment. Here we review the literature that links microglia to neural circuit remodeling and provide a discussion of the current understanding of how developmental alcohol exposure affects microglial behavior in the context of developing brain circuits.

## Introduction

The last decade has seen a renewed interest in microglial roles in the brain. This has been spurred by new molecular and imaging technologies that have allowed scientists to explore the functions of these cells within intact neural tissue throughout the lifespan. While classically thought of as immune cells that originate outside of the nervous system and have little to contribute to normal brain activities, recent research has painted a picture of microglia as complex brain cells that provide important contributions during both pathological and physiological conditions. Because microglia enter the brain early in development, they have the potential to influence the development of neural networks. Here we review the literature that has illuminated microglial contributions to the development of neural cells and their connectivity. We also discuss how microglial behavior could contribute to alterations in neural network remodeling in the context of fetal alcohol spectrum disorder (FASD), a neurodevelopmental disorder caused by gestational exposure to ethanol that results in devastating sensory and cognitive deficits.

## Microglial Roles in The Development of Neural Networks

### The Origin of Microglia

Microglia, the resident immune cells of the central nervous system (CNS), were first identified and documented by Pio del Río Hortega, who made several key observations based only on his histological studies (Del Río Hortega, 1920). Impressively, many of these observations have been confirmed with modern techniques. Later histological studies described macrophages localized in the CNS and then classified these migratory brain macrophages as microglia based on their morphological similarities to the cells described by Hortega (Perry et al., 1985). While many similarities exist between microglia and macrophages, these are now known to be separate populations of cells and microglia display different phenotypes in the unperturbed brain as compared to peripheral macrophages based on surface marker comparisons (Carson et al., 1998).

Microglia are derived from a unique population of primitive yolk sac myeloid progenitors and begin populating the brain around embryonic day 9.5 (E9.5) in the mouse (Kierdorf et al., 2013), a process that is dependent on microglial colony stimulating factor-1 receptor expression (CSF-1R; Alliot et al., 1999; Ginhoux et al., 2010). Microglial infiltration of the CNS is a complex multistep process in which microglia first accumulate on the pial surface before colonizing the parenchyma (Swinnen et al., 2013). Prior to birth, microglia represent only 1.7% of the CNS cell population and thus more than 95% of the adult microglial cell population is produced during early postnatal development and after the closure of the blood brain barrier (Alliot et al., 1999). The cortical colonization process involves three steps that occur from E10.5 to E17.5 (Swinnen et al., 2013). At first there is a slow increase in microglial cell numbers in the meninges due to proliferation from E10.5 to 14.5. This is followed by a transient rapid phase of population growth likely involving both infiltration and proliferation. During this phase microglia are recruited towards the ventricular and subventricular zones (SVZs) at E14.5 by basal progenitors which secrete the chemokine CXCL12 (Arno et al., 2014). The final phase is slower with reduced microglial proliferation and the beginning of microglial process formation. Beginning at E14.5, microglia begin to extend and retract their processes thereby surveying the CNS parenchyma during colonization. From E15.5 on, microglia are found near the ventricular and intermediate zones of the cortical wall (Swinnen et al., 2013). Interestingly, the proliferation and survival of developing microglia and basal progenitors appears to be tightly linked. A reduction in basal progenitor populations greatly reduces cortical microglia (Arno et al., 2014), while loss of microglial CSF-1R decreases the progenitor pool. Once the CNS matures, adult microglia show little turnover or proliferation and in the absence of pathological events, haematopoietic progenitors do not significantly contribute to microglial homeostasis (Ajami et al., 2007; Ginhoux et al., 2010). However, loss of CSF-1R signaling can induce apoptosis in microglia throughout the lifespan and specific progenitor populations with highly proliferative transcriptome profiles can rapidly generate microglia within the brain in response to microglial loss (Elmore et al., 2014).

In the healthy adult brain, unperturbed microglia are highly ramified and have highly motile processes. Early *in vivo* imaging studies, enabled by the generation of a transgenic mouse which selectively expresses enhanced green fluorescent protein (EGFP) in microglia under the fractalkine receptor *Cx3cr1* gene locus (Jung et al., 2000), estimated that these dynamic microglial processes were capable of surveying the entire brain parenchyma in a couple of hours (Davalos et al., 2005; Nimmerjahn et al., 2005). While this dynamic behavior could be related to microglial immune functions, more and more studies have implicated microglia as contributing to processes occurring in the absence of brain pathology. Certainly, these unperturbed microglia have a unique transcriptome profile with high levels of expression of *Cx3cr1, Trem2, P2ry12, Tgfβ1, Tgfβr1* and *Hexb* when compared to peripheral macrophages (Hickman et al., 2013; Butovsky et al., 2014), suggesting that they have functions distinct from their immune repertoire. Even after microglial depletion, repopulating microglia still maintain these distinct microglial transcriptome profiles, albeit with additional proliferative markers expressed during early repopulation (Elmore et al., 2014). The maintenance of a microglial profile even after robust depletion and repopulation suggests that there is a fundamental importance of microglial gene expression to normal brain function. Thus, infiltrating peripheral macrophages with differentially expressed genes will likely fail to fully serve the functions of microglia present in the CNS, thereby contributing to disease progression and failed maintenance of CNS homeostasis. As unique markers become evident from transcriptome analyses, we begin to gain a better understanding of the functional differences between macrophages and microglia. These differences can then aid in identifying microglial specific functions with relevance to neural cell interactions and the unique roles that microglia play in neurodevelopment and adult brain function. Recently, such roles have begun to be described, and an emerging consensus suggests that microglia are critical for the appropriate development of neurons and their synaptic connections.

### Microglia Regulate Neuronal Populations in the Embryonic Brain

**Figure 1:**
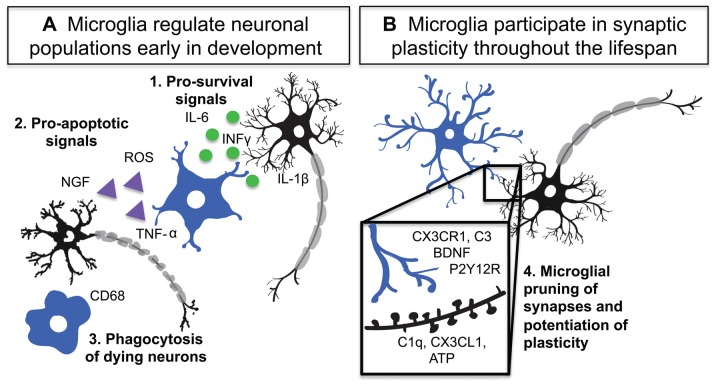
Microglia are active participants in neuronal circuit development and maintenance. **(A)** Microglia regulate neuronal populations through: **1**. Release of pro-survival signals such as interleukin-1 beta (IL-1β), interferon-gamma (INF-γ) and interleukin-6 (IL-6), **2**. Activation of Cd11b and DAP12 as well as release of reactive oxygen species (ROS) and tumor necrosis factor alpha (TNF-α) by microglia lead to neuronal apoptosis **3**. Phagocytosis of dying neurons and debris by microglia. **(B)** During development and adulthood microglia participate in synaptic plasticity and pruning through numerous signaling mechanisms including: **4**. Complement system driven phagocytosis of synapses, CX3CR1 mediated regulation of circuit development, P2Y12 receptor (P2Y12R) dependent synaptic plasticity, and microglial release of brain derived neurotrophic factor (BDNF).

During the development of the nervous system there is an overproduction of neural cells, which are then systematically eliminated to generate the adult cell distribution and connectivity of the CNS (Yuan et al., 2003). This process is critically important in the generation of mature circuitry. Microglia as innate immune cells have the capability to induce controlled cell death in pathogens (Ransohoff and El Khoury, 2015) and as early infiltrators of the CNS are present at the right time to contribute upstream pro-apoptotic signals to neurons (Ginhoux et al., 2010; Swinnen et al., 2013; Arno et al., 2014). Thus far, multiple groups have seen that microglia utilize various signaling mechanisms to participate in neural cell population regulation and phagocytosis (Figure 1; Frade and Barde, 1998; Marín-Teva et al., 2004; Sedel et al., 2004; Peri and Nüsslein-Volhard, 2008; Wakselman et al., 2008).

#### Trophic Support

One important facet of neural development is the presence of trophic or pro-survival signals, which support the generation of neuronal populations. In both the developing macaque and rat brain, microglia exist in close proximity to populations of proliferating neurons in the SVZ (Cunningham et al., 2013). Microglia provide numerous pro-survival signals during development. In the postnatal rat SVZ from postnatal day 1 through 10 (P1–P10), microglia are densely populated and promote both neurogenesis and oligodendrogenesis (Shigemoto-Mogami et al., 2014). Inhibition of microglia with minocycline decreases microglial activation with concomitant decreases in the expression of nestin and Ki67 demonstrating that microglial activation status is vital to proliferation of SVZ progenitors. These activated microglia provide optimal levels of interleukin 1-beta (IL-1β), interferon-gamma (INF-γ), and interleukin-6 (IL-6) to stimulate both neurogenesis and oligodendrogenesis in the SVZ. Microglial population levels in the white matter peak at P3-P7 and they impact subcerebral and callosal axonal projections (Ueno et al., 2013). Elimination of microglia significantly increases layer V cortical neuron apoptosis in a microglial CX3CR1 and insulin growth factor-1 (IGF-1)-dependent manner. In the absence of microglial CX3CR1 signaling or in the presence of minocycline, IGF1BP (IGF-1 binding protein) expression increases and therefore IGF-1 support available to neurons decreases (Ueno et al., 2013). Together these studies show that microglia provide important signals for proliferation and survival of neurons in multiple brain regions during early development of the CNS.

#### Cell Death

Appropriate induction of cell death is as important as tropic support for normal CNS development. Interestingly, some typical pro-survival signals can serve pro-apoptotic roles during development as well. In the developing chick retina, microglial nerve growth factor (NGF) is vital to neural apoptosis (Frade and Barde, 1998). During development, microglia provide NGF to promote cell death through the neuronal p75trka receptor. CD11b and DAP12 also serve as microglial pro-apoptotic signaling mechanisms (Wakselman et al., 2008). At P0, approximately 50% of apoptotic neurons in the hippocampus are in contact with microglial processes. Microglia in the mouse subiculum and a portion of CA3 express CD11b and DAP12 during hippocampal development. Mice deficient in either CD11b and/or DAP12 signaling have significant reductions in neural apoptosis though the effects are not additive, suggesting that the receptors act within the same pathway. The consequence of losing these signals is a loss of reactive oxygen species (ROS) production leading to decreased neuronal apoptosis. Similarly, in the cerebellum Purkinje cells expressing activated caspase-3 are surrounded by amoeboid microglial processes (Marín-Teva et al., 2004). Ablating microglia using clodronate or blocking microglial ROS bursts using diphenyleneiodonium rescues Purkinje cells during development. Comparable mechanisms exist in the spinal cord. By E13, motor neurons are committed to cell death by extrinsic factors (Sedel et al., 2004). Interestingly, spinal explants with absent macrophage populations fail to undergo programmed cell death. Mesenchyme conditioned media can also induce apoptosis in the motor neurons supporting the hypothesis that a diffusible factor is the death signal. This signal appears to be tumor necrosis factor alpha (TNF-α), which is produced by macrophages at E12, since blocking TNF-α signaling specifically at E12, but not E13 blocks apoptosis. This finding demonstrates that macrophages specifically commit motor neurons to cell death within a specific window of development (Sedel et al., 2004).

#### Phagocytosis

During development it is also essential that the debris from dying and dead cells is efficiently cleared to permit survival of remaining cells. Microglia are vital participants in this process as they are capable of rapid clearance of damaged and dying cells (Sierra et al., 2013). Microglia of the SVZ phagocytose both Tbr2+ and Pzx6+ neural progenitors to varying degrees from E50 to E100 in macaques (Shigemoto-Mogami et al., 2014). In the mouse, the microglia of the SVZ maintain an amoeboid shape, which is often tied to phagocytic activity, until about P14 when neurons are maturing and cell death has ended. They also express CD68 at levels that are much higher than those seen in older animals. When minocycline is administered to inhibit microglial activation, the microglia of the SVZ show increased ramification and decreased CD68 labeling (Shigemoto-Mogami et al., 2014), suggesting that this early developmental state is reminiscent of immune activation and phagocytosis later in life. Microglia also maintain the ability to phagocytose dying neurons later in life. In the subgranular zone of the adult hippocampus, microglia efficiently engulf 90% of apoptotic cells (Sierra et al., 2010).

Taken together these studies implicate microglia as key regulators of developing neuronal populations. Interestingly, across these varied regions, microglia are integral in supplying not just pro-apoptotic signals, but also survival signals. Thus, a perturbation of microglial dynamics during embryonic and early postnatal development could have profound effects on the neuronal landscape of the CNS.

### Microglia Contribute to the Remodeling of Developing Circuits

After neuronal populations are established and developmental apoptosis is complete, neurons generate circuits by forming and remodeling synapses. During their exploration of the brain parenchyma, microglial processes make frequent contacts with excitatory synaptic elements, including both axon terminals and dendritic spines (Tremblay et al., 2010). These contacts can be modified by both injury and sensory induced changes in experience (Wake et al., 2009; Tremblay et al., 2010). Neuronal activity may be key to modulating microglial contacts with synapses as it can affect process motility. In the retina, increasing ionotropic glutamatergic transmission increases microglial motility, while increasing ionotropic GABA-ergic transmission slows microglial dynamics (Fontainhas et al., 2011). The effects are not cell autonomous and are instead mediated by putative release of purines by neurons as their activity changes. In the zebrafish, microglia are steered towards active neurons also through neuronally-released extracellular signals (Li et al., 2012). Similarly, in the cortex, blocking GABA-ergic transmission increases microglial sampling, and in acute hippocampal slices repeated NMDAR activation can trigger microglial process outgrowth through ATP release (Dissing-Olesen et al., 2014). During hippocampal long term potentiation (LTP) induction, microglia increase their process number and contacts made with synapses (Pfeiffer et al., 2016).

While it has yet to be determined whether changes in microglial motility impact how microglia interact with synapses, there are several pieces of evidence suggesting this may in fact be the case. *In vivo*, microglia preferentially make putative contacts with small spines which are frequently eliminated (Tremblay et al., 2010). Because small spines tend to represent weaker synapses with lower levels of activity (Matsuzaki et al., 2001), this suggests that microglia may have a preference for synapses with different activity patterns. Sensory modification in the form of dark rearing during adolescence results in increased dendritic spine motility and turnover in the primary visual cortex (Tropea et al., 2010). This same manipulation reduces microglial motility and changes microglial preference to larger spines. These spines shrink upon microglial contact in a manner akin to long-term depression (LTD; Zhou et al., 2004; Tremblay et al., 2010). A different manipulation, monocular deprivation, in which only the contralateral eye is deprived, but evoked activity in the visual cortex is also decreased, also decreases microglial motility, suggesting that *in vivo*, reductions in neuronal firing or synaptic events may slow microglial dynamics. Although microglia-spine interactions were not examined dynamically in this study, monocular deprivation did increase the association between microglial processes and synaptic clefts (Sipe et al., 2016). A pioneering study also examined microglial actions at synapses in pathological settings. During transient cerebral ischemia there is an injury-associated reduction in neuronal activity, and microglia target and contact spines which are later eliminated (Wake et al., 2009). In ischemic conditions, microglial contacts are prolonged, and it appears as though the terminal ends of the processes wrap around the synaptic structures that are later eliminated. The ability of microglia to engulf ischemic terminals suggests that microglia could also utilize this phagocytic process to eliminate synaptic elements during plasticity.

Microglial regulation of both synaptic outgrowth and pruning is an important component of their participation in plasticity and neural circuit development (Figure 1). A number of studies have indicated that microglia are active participants in developmental and adult plasticity and that during these processes microglia make dynamic contacts with synaptic elements and promote elimination or growth of dendritic spines (Tremblay et al., 2010; Schafer et al., 2012; Parkhurst et al., 2013; Zhan et al., 2014; Pfeiffer et al., 2016; Sipe et al., 2016). In the earliest stages of synaptic development, microglia may be critical for the initiation of postsynaptic specializations. In the barrel cortex, microglia influence the formation of transient filopodial protrusions from P8 to P10 in the mouse (Miyamoto et al., 2016). This effect of microglial contact with the dendrite does not extend to later ages. Interestingly, microglia are amoeboid and activated during this stage of development, suggesting that this activation state may be important to barrel cortex synaptogenesis (Miyamoto et al., 2016). After initial synaptic synaptogenesis, excessive and inappropriate synapses must be pruned back to generate functional circuits. This process is usually dependent on sensory-driven activity in neurons, and microglia appear to play an important role. For instance, in the early development of the retinogeniculate system, microglia help prune away extraneous synapses in the lateral geniculate nucleus (LGN) based on the activity of retinal ganglion cells in the eye (Schafer et al., 2012). Microglia appear to be able to remove synapses through phagocytosis with great specificity—limiting engulfment to terminals coming from less active retinogeniculate cells. Microglia also infiltrate the barrel cortex and hippocampus during early postnatal development and promote pruning of excess synapses; failure of infiltration results in a transient hyperconnectivity which normalizes, presumably through non-microglial specific mechanisms, in adulthood (Paolicelli et al., 2011; Hoshiko et al., 2012; Arnoux et al., 2013). Consistent with this, removing and adding microglia to hippocampal organotypic slices results in increased and decreased synaptic numbers, respectively (Ji et al., 2013). While these effects are carried out by immature microglia, which are still amoeboid and express phagocytic markers, it is unclear whether microglia contribute to synaptic remodeling in their adult ramified state. A recent study showed that ramified microglia in the adolescent mouse visual cortex were highly attuned to plastic changes in neurons, undergoing rapid changes in morphology, dynamics, phagocytic profiles and contacts with synapses when neurons are remodeled (Sipe et al., 2016). Removing a single purinergic receptor from microglia disrupts synaptic plasticity, suggesting that these changes are critical for circuit rewiring (Sipe et al., 2016). In the adult mouse only, one study has shown that microglial depletion results in a loss in motor learning-dependent synapse formation in the motor cortex (Parkhurst et al., 2013). This effect appears to be dependent on microglial brain-derived neurotrophic factor (BDNF) production. While this mechanism was found in the adult, the presence of trophic factors and microglia during many developmental stages suggests that BDNF could be a key player in regulating microglial synapse outgrowth. It is important to note that another study that depleted microglia in the adult using a different method did not find effects on motor learning (Elmore et al., 2014), but temporally complex effects of microglial depletion on both spatial learning and sociability in adulthood have been described (Torres et al., 2016). Thus, the role of microglia in adult synaptic plasticity is still controversial and may be more complex than microglial effects during development.

### Recycling of Neuroimmune Molecules for Synaptic Sculpting

While it is becoming clear that microglia can contribute to synaptic plasticity in development and beyond, little is known about the molecular repertoire used by physiological microglia to implement these functions. The few mechanisms that have been described suggest that immune signaling is critical in the normal brain and that microglia may recycle their immune signaling capabilities to implement their roles in the physiological brain (Figure 1).

#### Complement Cascade

An important immune signaling system implicated in microglial mediated synaptic plasticity in the developing brain is the complement cascade (Stephan et al., 2012). In pathological conditions the complement cascade is involved in the tagging and clearance of pathogens (Sierra et al., 2013). *In vitro*, microglia exposed to complement 5a (C5a) show rapid G-protein driven actin cytoskeleton rearrangements in order to produce membrane ruffling and quickly extend processes (Nolte et al., 1996). Mice deficient in either C1q, the initiation protein, or downstream C3 show a lack of synapse elimination and immature connectivity between the retina and the LGN (Stevens et al., 2007). They also lack microglial engulfment of axonal terminals suggesting that microglia use this pathway to phagocytose synapses. In this case, C1q is produced by neurons specifically during the period of synaptic pruning, although it is not yet known how specific synapses are tagged for elimination by the complement system. The production of neuronal C1q is dependent upon astrocytic transforming growth factor β (TGF-β); mice lacking TGF-β phenocopy the deficiencies seen in the retinogeniculate system from loss of C1q or C3 (Bialas and Stevens, 2013). This shows that developmental synaptic pruning involves the interplay of astrocytic, microglial and synaptic activities that converge on a neuroimmune pathway that remodels neuronal circuitry. While complement is vital for the involvement of microglia in the developing retinogeniculate system, it can also become reactivated during disease (Hong et al., 2016). In mice modeling Alzheimer’s disease, prior to plaque formation, there is a notable loss of synapse density. Accompanying this pre-plaque loss of synapses is an increase in C1q and microglial phagocytosis. Thus, a mechanism of synapse regulation from development can become pathological when reactivated later in life, leading to progression of a neurodegenerative disease such as Alzheimer’s (Hong et al., 2016). Furthermore, a recent landmark study demonstrated through elegant *in vitro* and *in vivo* experiments that astrocyte activation in response to lipopolysaccharide exposure depends on the release of C1q, TNF-α, and IL-1α from microglia, indicating that microglial activation is indeed an initial trigger to which other glial cells react and become neurotoxic (Liddelow et al., 2017).

#### Fractalkine Signaling

Fractalkine has also arisen as a key microglial signal during nervous system maturation. Fractalkine (CX3CL1) is a chemokine which uniquely exists in both a membrane bound and secreted form and is expressed constitutively by neurons (Harrison et al., 1998). The fractalkine receptor (CX3CR1) consists of a CX3C motif and a mucin-like stalk which anchors it to the cell membrane (Jung et al., 2000) and within the CNS, microglia are the only cells that express this receptor (Harrison et al., 1998). Constitutive release of fractalkine is thought to keep microglia quiescent and loss of the receptor results in augmented responses to injury and insult (Jung et al., 2000; Cardona et al., 2006; Corona et al., 2010). Heterozygous CX3CR1/green fluorescent protein (GFP) knock-in mice (CX3CR1 Het) are used extensively to label microglia for *in vivo* two-photon imaging studies; microglia are selectively and efficiently labeled through a disruption mutation replacing one copy of CX3CR1 with GFP (Jung et al., 2000). Due to the expression patterns of the ligand and receptor, and the extensive use of this transgenic mouse for imaging, it is critical to understand how the fractalkine receptor contributes to synapse reorganization.

Multiple studies have shown that CX3CR1 null mice have deficits in circuit development and remodeling. These mice have deficits in memory formation that are accompanied by a gene-dependent overexpression of both IL-1β and p38 (Rogers et al., 2011) and exhibit impaired social behavior (Zhan et al., 2014). Additionally, the normal plasticity boosting effects of environmental enrichment are also lost in animals lacking CX3CR1 (Maggi et al., 2011). On a synaptic level, loss of fractalkine signaling results in an overabundance of immature synapses (Paolicelli et al., 2011). This deficit is transient, as synapse number returns to normal by early adulthood. CX3CR1 signaling in the barrel cortex appears to have a local effect of recruiting microglia to developing thalamocortical synapses where they then influence the AMPAR/NMDAR ratio at these synapses (Hoshiko et al., 2012). Impairment of fractalkine receptor function also leads to immature synaptic multiplicity (Zhan et al., 2014). Synaptic multiplicity is the presence of multiple synapses on the same axonal terminus. In CX3CR1 null animals, there is a failure to form multiple synapses, which is indicative of immature connectivity in the neural network. However, it is unclear whether fractalkine signaling is directly involved in mediating microglia-synapse interactions in these studies. Other studies show that in CX3CR1 null mice, there is a loss of adequate developmental migration of microglia within the hippocampus (Paolicelli et al., 2011) and into the barrels in somatosensory cortex (Hoshiko et al., 2012). Thus, deficits in synaptic pruning may be a result of the reduced number of microglia within the tissue where these synapses are located rather than fractalkine signaling *per se*. CX3CR1 deficient mice also show a delay in the appearance of microglial cells expressing the voltage-gated potassium channel Kv1.3 in barrel cortex (Hoshiko et al., 2012; Arnoux et al., 2013). This transient channel expression could be important for the invasion of microglia into barrels or into the parenchyma in general.

#### Purinergic Signaling

In their quiescent state, microglia express the purinergic receptor P2Y12 at very high levels (Sasaki et al., 2003; Hickman et al., 2013; Butovsky et al., 2014), and this expression appears to be very specific to microglia as opposed to other cells in the brain (Zhang et al., 2014). P2Y12 is a Gi-coupled receptor which generates microglial chemotactic response to ATP and focal injury (Davalos et al., 2005; Haynes et al., 2006). The receptor is down-regulated by injury, but highly expressed by microglia in the healthy brain (Haynes et al., 2006), possibly because its role is limited to the very early injury response. However, this places signaling through this receptor as a possible candidate for mediating microglial physiological functions. Removing P2Y12 signaling, either genetically or pharmacologically, abolishes ocular dominance plasticity, an experience-dependent process that occurs in the visual cortex during adolescence (Sipe et al., 2016). Along with a lack of plasticity, lack of P2Y12 signaling also abolishes the microglial responses that likely underlie this plasticity, including changes in morphology, dynamics, phagocytosis of synaptic material and physical contacts with the synaptic cleft. The loss of functional plasticity, and concomitant microglial changes in the absence of P2Y12 signaling suggest that microglia actively participate in this form of plasticity and that purinergic signaling is critical to this process. While it is unclear where the purinergic signal originates and what mechanisms lead to P2Y12 receptor activation on microglia, several lines of evidence suggest that purines may be released by neurons in an activity-dependent fashion. In acute brain slices, ATP is released during stimulation of NMDA receptors, and this ATP is sufficient to activate microglial P2Y12 and generate chemotactic responses in the absence of injury (Dissing-Olesen et al., 2014). Similar effects have been observed in pathological conditions where purinergic signaling is tied to changes in phagocytic potential (Eyo et al., 2014; Abiega et al., 2016). Neuronal release of purines is also thought to be responsible for changes observed in microglial dynamics after manipulation of neuronal activity in the retina (Fontainhas et al., 2011). Additionally, during LTP in the CA1 region of the hippocampus, NMDAR activity is essential to increases in the duration of microglial contacts with spines (Pfeiffer et al., 2016). LTP over time leads to structural changes in spines, and increased microglial contact time suggests that microglia could participate in stabilizing spine outgrowth. Thus, purinergic release from neurons could contribute to synapse phagocytosis and synaptic outgrowth by signaling to microglia.

Microglia play an important role during synaptogenesis and synaptic pruning and as such their functions are likely critical both to normal development and when this process goes awry in neurodevelopmental disorders. The highly dynamic movement of microglial processes is key to their activity at synapses. The very apparent response of microglia to changes in synaptic homeostasis implies that these serve an instrumental role in establishing neural circuitry during periods of robust plasticity during development. Indeed, a number of microglial signaling mechanisms have already been implicated in the developmental wiring of the CNS (Paolicelli et al., 2011; Schafer et al., 2012; Sipe et al., 2016). The ability of microglia to release various cytokines as well as important growth factors means that they can diversely impact neuronal wiring depending on the unique milieu and processes of different regions of the CNS. While this field may still be growing, it is becoming increasingly clear that microglia sense and respond to neural activity with chemotactic process movement (Fontainhas et al., 2011; Li et al., 2012; Dissing-Olesen et al., 2014; Pfeiffer et al., 2016). These movements in turn permit microglia to contact and potentially provide important factors to neurons, which may then influence the production or elimination of synaptic structures. The exact nature of microglial-neuron interfacing during process contact remains to be elucidated, and future work will hopefully uncover clear mechanistic explanations for the phenomenon observed to date. Interestingly, perturbing the microglial population during embryonic development results in a robust over production of dopaminergic inputs into the subpallidum as well as abnormalities in neocortical inhibitory tone (Squarzoni et al., 2014), suggesting that microglial dysfunction could lead to profound effects on neurodevelopment. In fact, microglial dysfunction has already been implicated in a number of neurodevelopmental disorders including Rett syndrome (Derecki et al., 2012; Cronk et al., 2015), autism (Zhan et al., 2014) and schizophrenia (Sekar et al., 2016). From the emerging evidence, it appears that microglia may be serving widespread important roles in CNS circuitry development, though more investigation will be necessary to better assess the extent of microglial participation in neuronal circuit formation. In the remainder of this review article we will focus on synaptic deficits in FASD and discuss the role microglia could play in alcohol-induced circuit dysfunction.

## Fetal Alcohol Spectrum Disorder

FASD is the leading cause of non-heritable mental disability in the United States, estimated to affect 2%–5% of live births (May et al., 2014). Unfortunately, alcohol consumption during pregnancy remains common in the U.S. with 1 in 10 pregnant women reporting any level of alcohol consumption and 1 in 30 pregnant women reporting binge levels (four or more drinks in one occasion) of alcohol consumption in the last 30 days (Tan et al., 2015). No amount of alcohol consumption during pregnancy is considered safe (Sokol et al., 2003), though more frequent drinking, consuming drinks with higher alcohol concentration, and binge drinking are all associated with a higher risk of FASD and severity of FASD symptoms in offspring (Feldman et al., 2012; Avalos et al., 2014; May et al., 2016). The most severe consequences of gestational EtOH exposure are caused by the overt toxicity of EtOH, leading to microcephaly (small brain size) resulting in profound mental disability, thought to be caused in part by EtOH-induced widespread neuronal cell death (Clarren et al., 1978; Ikonomidou et al., 2000). The underlying mechanisms by which more moderate gestational EtOH exposure causes cognitive impairment is still not clearly understood. FASD patients often suffer from attention difficulties and impulsivity, misinterpretation of social cues (Stevens et al., 2012; Kerns et al., 2016), learning disabilities and impaired memory (Streissguth et al., 1994a,b; Furtado and Roriz, 2016), aggression (Sood et al., 2001), inappropriate sexual behavior (Streissguth et al., 2004), underdeveloped fine motor skills (Kalberg et al., 2006), and sensory processing deficits, including visual (Vernescu et al., 2012; Doney et al., 2016) and auditory (Stephen et al., 2012) information processing.

Many of the cognitive symptoms impaired in FASD, including learning and memory, are known to depend on normal neuronal network development and plasticity, specifically the ability of neuronal networks in the brain to remodel their structure and function as a result of changes in environment or experience. This suggests that after developmental EtOH exposure, the ability of the brain to undergo appropriate developmental plasticity is impaired. Understanding how synaptic structure and plasticity is impaired by gestational EtOH exposure and the role of neurons and glia in this process is critical for developing future treatments for FASD. In fact, there is ample evidence that developmental EtOH exposure has widespread effects throughout the brain and can affect the developmental trajectory of many different cell types in different brain areas, depending on the severity and timing of exposure. As discussed in detail below, neurons can be affected acutely by EtOH causing neuronal cell death, and long-term by changes in the function and synaptic connections of those neurons that survive. For example, EtOH-dependent effects on the structure and function of radial glial cells (Shetty and Phillips, 1992; Vallés et al., 1996) likely contribute to changes in neuronal migration and development. Additionally, changes in radial glial cells can alter astrocytic function, as radial glia give rise to astrocytes (Miller and Robertson, 1993). Because astrocytes provide critical cues for neuronal development, EtOH-induced changes in the number and behavior of developing astrocytes can have profound effects on neuronal structure and the connectivity of nascent neuronal networks (Tomás et al., 2005; Giordano et al., 2011; Paul and Medina, 2012). Additionally, while not very thoroughly studied to date, oligodendrocytes may be sensitive to developmental EtOH exposure (Creeley et al., 2013). EtOH-induced changes in oligodendrocyte number and the reprogramming of surviving oligodendrocyte precursor cells could lead to reductions in myelination that can have large effects on brain function (Pinazo-Duran et al., 1997; Guerri et al., 2001; Sowell et al., 2008).

EtOH exposure has also been linked to inflammatory processes both in the developing and adult brain. Thus potential link between EtOH-induced changes in microglial behavior and EtOH-induced changes in synaptic structure and plasticity is an area of great current interest. Here, we will review what is presently understood about microglial contributions to FASD, addressing developmental EtOH’s effects and/or potential effects on the spectrum of microglial functions critical for normal brain function and development.

## Microglial Roles in FASD

### Developmental EtOH Exposure as a Trigger for Microglial Immune Functions

**Figure 2:**
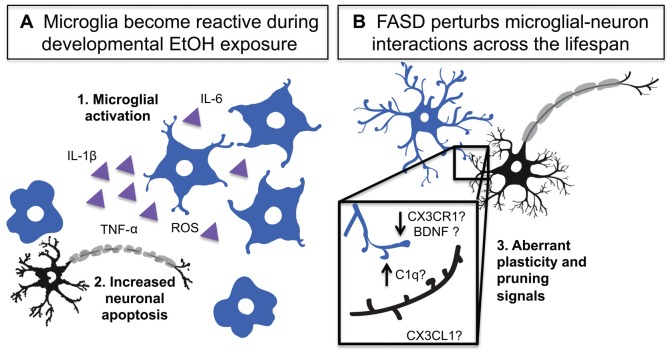
Developmental EtOH exposure activates microglia and impairs their homeostatic functions. **(A)** Developmental EtOH exposure causes acute microglial activation which produces: **1.** Activation and production of inflammatory signals **2.** Increased neuronal death and phagocytosis. **(B)** Developmental EtOH exposure produces changes in dendritic spine density and could impact microglial interactions with synapses. **3.** A number of microglia-neuron signaling systems could be disrupted and contribute to spine density changes including: complement dependent pruning, decreased CX3CR1 signaling altered BDNF release.

The potential for developmental EtOH to cause changes in microglial function, either acutely or long-term has only recently become a focus of intense research. This is an attractive mechanism for EtOH’s effects on developing circuits because of the pluripotent roles microglia play in development and adult brain function as described above and because their immune status makes them likely to be sensitive to EtOH exposure (Figures 1, 2). Additionally, because these cells are generated in the yolk sac during early development and exhibit low turnover once they mature in the brain, EtOH-exposed microglia may persist in the brain throughout life and thus alter brain function long-term. The major hypothesis propelling the majority of current research is that EtOH causes a dysregulation of the immune functions of microglia, leading to a widespread pro-inflammatory environment that is perpetually harmful to neurons and fails to resolve with age (as reviewed by Block et al., 2007; Guizzetti et al., 2014; Kane and Drew, 2016). Part of this hypothesis has been born out in a series of studies showing that changes in the immune functions of microglia can be acutely triggered by high binge levels of EtOH exposure at multiple life stages, not only during the brain growth spurt (BGS) when synapses are initially being formed (Dobbing and Sands, 1979), but also during adolescence and adulthood (as reviewed by Drew and Kane, 2014). However, long-term changes in microglial function have not been easily demonstrated, suggesting that if EtOH alters microglia chronically, the effects may be more subtle than perpetual inflammatory signaling.

The BGS studies collectively show that within 24 h after the last EtOH dose, microglia in certain regions of the cerebellum (Kane et al., 2011; Drew et al., 2015; Topper et al., 2015), parietal and somatosensory cortices (Ahlers et al., 2015; Drew et al., 2015), and hippocampus (Drew et al., 2015; Boschen et al., 2016) show morphological signs of activation (Figure 2). Morphological signs of microglial activation that have been reported in rodent FASD models include increased prevalence of amoeboid microglia, decreased microglial process spread, decreased microglial process ramification, and increased process thickness (Kane et al., 2011; Drew et al., 2015; Topper et al., 2015; Boschen et al., 2016). Aside from morphological changes, microglial gene expression is also altered in a way that is consistent with immune activation: Iba-1 expression increases in hippocampus, cerebellum and parietal cortex (Drew et al., 2015), and P2Y12 expression has been shown to decrease in morphologically activated microglia in layers II and IV of the somatosensory cortex (Ahlers et al., 2015). This is accompanied by increased pro-inflammatory as well as anti-inflammatory cytokine/chemokine production, namely IL-1β, TNF-α, MCP-1, TGF-β and IL-10 (Ahlers et al., 2015; Drew et al., 2015; Topper et al., 2015; Boschen et al., 2016), likely produced not only by immunologically activated microglia but also by activated astrocytes (Topper et al., 2015), which also produce these factors.

It is important to note that studies have not always observed microglial activation after developmental EtOH exposure in every brain region examined. In one study of P10 rat hippocampus after P4–P9 high binge EtOH exposure, sham intubation and EtOH intubation were both shown to increase microglial activation in the dentate gyrus to approximately the same degree, compared to suckle control (Boschen et al., 2016). However, in CA1 and CA3, the EtOH intubated animals showed morphological signs of microglial activation above that of both the suckle control and sham intubated control (Boschen et al., 2016). In a second recent study in which rat pups were exposed from P3 to P5 via EtOH vapor inhalation, changes in microglial morphology indicative of an immunologically activated state were much more robust in cerebellum, where Purkinje neuron cell death was also detected, compared to in hippocampus, where NeuN density was unchanged at all time points examined (Topper et al., 2015). Together, these results suggest that microglial contributions to network dysfunction after EtOH exposure are likely dependent on brain area. In fact, some studies have found that microglia are sensitive to their microenvironment, resulting in regional differences in density, distribution, morphology and expression patterns (Lawson et al., 1990; Vela et al., 1995; Arnoux et al., 2013). Additionally, a genome-wide analysis of microglia across multiple brain regions found that microglia exhibit region-dependent heterogeneity of transcriptional identities and such heterogeneous expression of both receptors and cellular effector molecules could explain region-specific reactions to perturbations such as EtOH (Grabert et al., 2016).

Only a handful of studies have addressed microglial phenotype beyond 24–48 h after EtOH exposure, probing as far as 96 h (Ahlers et al., 2015), 22 days (Bodnar et al., 2016), and 40 days later (Topper et al., 2015). Their findings consistently suggest that microglial activation is not long-lived. All of these studies have shown that levels of pro-inflammatory cytokines and chemokines, including IL-1β and TNF-α, are no longer elevated at these later time points. Microglial morphology was investigated by Ahlers et al. (2015) and Topper et al. (2015) in somatosensory cortex and cerebellum respectively, revealing that microglia had recovered to a normally ramified phenotype. P2Y12 expression also increased to control levels (Ahlers et al., 2015). While more research needs to be done to determine whether prolonged microglial activation is present in specific brain areas, it appears that alterations in microglial function that are elicited by EtOH resolve rapidly or are more subtle than those that are evoked acutely.

### Direct and Indirect Causes of Acute Microglial Activation after EtOH Exposure

Multiple triggers are likely responsible for the acute activation response of microglia after developmental EtOH exposure. Microglia may directly respond to the presence of EtOH in the brain since they are exquisitely tuned to perturbations in the environment. Since EtOH is membrane permeable, it could diffuse into the microglial cytoplasm to modulate intracellular signaling or it could bind surface receptors on microglia that activate intracellular cascades leading to an inflammatory response. The involvement of toll-like receptor 4 (TLR4) signaling, a pathway particularly well understood in the context of microglial responses to pathogens and the activation of innate immune responses, has been studied in the context of microglial direct responses to EtOH. Cumulatively, studies suggest that in the adult, EtOH exposure can trigger microglial activation as well as proinflammatory cytokine and chemokine production (IL-1β, TNF-α, MCP-1) through downstream NF-κB activation (Qin et al., 2008; Qin and Crews, 2012; Crews et al., 2013). Such increases in proinflammatory cytokines and chemokines are dependent on TLR4 signaling (Fernandez-Lizarbe et al., 2009) and are not found in the absence of TLR4 activation (Alfonso-Loeches et al., 2010). While TLR4 is expressed in multiple cell types, *in vitro* studies involving BV2 microglia and *in vivo* studies involving rodents and/or examination of human postmortem tissue have shown that compared to neurons, microglia tend to express higher levels of TLR4 (Crews et al., 2013; Zhang et al., 2014; Lawrimore and Crews, 2017), suggesting that TLR4-mediated consequences of EtOH exposure may affect microglia more so than neurons. In human postmortem orbital frontal cortex, higher levels of TLR4 immunoreactivity correlated with higher levels of high mobility group protein B1 (HMGB1) immunoreactivity (Crews et al., 2013). HMGB1, in its secreted form, can bind TLR4 and enhance cytokine release as part of the damage associated molecular pattern (DAMP) that perpetuates inflammation. It appears that HMGB1 exacerbates microglial activation upon exposure to EtOH through its actions on TLR4 (Crews et al., 2013; Lawrimore and Crews, 2017). While it is unclear whether TLR4 is involved in EtOH responses during development, it is likely that developing microglia would be able to sense EtOH through a similar danger pathway. Additionally, developmental EtOH exposure has been shown to alter microglial cell number in at least one brain region. At P6 after oral EtOH exposure from P2 to P5, the number of microglia in the cerebellar vermis was reduced, suggesting that EtOH might be impairing microglial proliferation and/or leading to microglial cell death (Kane et al., 2011). While this effect could be driven by toxic signals produced by other injured cells, it seems likely that an effect on microglial cell number would be direct, as is EtOH induced neuronal cell death.

On the other hand, microglia are incredibly sensitive to perturbations to other cell types in the brain milieu, and thus could undergo immunological activation indirectly, due to EtOH-driven glial and neuronal injury and death. It is well established that high binge EtOH exposure, exceeding an estimated “toxic threshold” of 200 mg/dL in blood, can induce apoptotic neuronal cell death in many brain regions (Ikonomidou et al., 2000). These regions include prefrontal cortex (Olney et al., 2002), somatosensory cortex (Ahlers et al., 2015), visual cortex (Olney et al., 2002; Tenkova et al., 2003), hippocampus (Ikonomidou et al., 2000; Wozniak et al., 2004), retrosplenial cortex (Olney et al., 2002; Wozniak et al., 2004), anterior thalamic nuclei (Olney et al., 2002; Wozniak et al., 2004), mammillary bodies (Wozniak et al., 2004), and cerebellum (Goodlett and Lundahl, 1996; Light et al., 2002; Kane et al., 2011; Idrus and Napper, 2012; Topper et al., 2015). Additionally, EtOH exposure can affect glia causing reduced proliferation or cell death in astrocytes (Miller and Potempa, 1990), oligodendrocytes (Creeley et al., 2013) and microglia (Kane et al., 2011). In the context of neuronal cell death, the cerebellum and corresponding deficits in motor function have been particularly well studied by the FASD and general alcohol fields, as reviewed by Luo (2015). Multiple groups have demonstrated in rodents that moderate to high binge levels of EtOH exposure, even on a single day during the BGS, can lead to Purkinje cell apoptosis in the cerebellar vermis and lobules between 10 h and 24 h after exposure (Goodlett and Lundahl, 1996; Light et al., 2002; Kane et al., 2011; Idrus and Napper, 2012; Topper et al., 2015). Loss of Purkinje cells is also evident later in life, suggesting that these neurons are truly lost. However, the reductions seen in EtOH compared to control animals tend not to be as drastic as expected given the widespread apoptotic response seen at earlier time points (Idrus and Napper, 2012; Topper et al., 2015). Still, EtOH-induced loss of these critical inhibitory neurons, which integrate the output for motor function, leads to lasting deficits in gait patterns, an indication of impaired motor coordination (Topper et al., 2015). The death of neurons could have a profound effect on microglial activation during the time of EtOH exposure.

The indirect effects of EtOH-induced neuronal death on microglial activation have recently been demonstrated in an elegant *in vivo* study. Microglial activation was observed only in layers of somatosensory cortex with cleaved caspase-3 positive or PSVue positive cell bodies, leading these researchers to hypothesize that the microglial activation response could be caused by apoptotic cell death (Ahlers et al., 2015). They utilized mice genetically inhibited from undergoing apoptosis (BAX knockout mice) and found no morphological changes in microglia, no changes in P2Y12 expression, and no production of IL-1β or TNF-α at any time point (4–96 h) after EtOH exposure when neurons were not dying (Ahlers et al., 2015). This suggests that microglial activation and the onset of neuroinflammation is not occurring in response to EtOH itself, but in response to the apoptotic cell death caused by the EtOH. These findings also indicate that an inflammatory microglial contribution to FASD pathology is likely limited to the early period of EtOH exposure and that long-term effects on circuit function propagate from neuronal injury at that earlier time point.

There is also evidence that the proinflammatory environment itself can underlie at least some of the cellular damage and death caused by developmental EtOH exposure. Preemptive and concurrent administration of peroxisome proliferator-activated receptor-γ (PPAR-γ) agonists has an anti-inflammatory effect and prevents BGS EtOH-driven microglial activation (Kane et al., 2011; Drew et al., 2015). This dampening of the otherwise pro-inflammatory environment in turn ameliorates the reductions in both microglial and Purkinje cell number in the cerebellum (Kane et al., 2011). These findings suggest that while neuronal cell death induces microglial activation, microglial activation may in turn promote conditions that lead to increased neuronal death. PPAR-γ agonism is also effective at decreasing microglial activation in response to developmental EtOH exposure in other brain regions, including hippocampus and cerebral cortex (Drew et al., 2015).

Together the studies highlighted in this section support the overall conclusion that microglial activation is secondary to the presence of apoptotic cell bodies, but that the factors expressed in or released from apoptotic cell bodies could have a direct effect on microglial intrinsic signaling that in turn continues to support an immunologically activated microglial state. At the same time, it has become increasingly clear that microglia do not remain in an activated state for long, resolving quickly to what appears to be a physiological state within days after initial EtOH exposure (Ahlers et al., 2015). Mechanisms by which this resolution in microglial phenotype occur constitute a new frontier in FASD research, while potential ways in which the actual ability of microglia to complete their physiological functions long-term after developmental EtOH exposure will be discussed in reviewed above.

### Effects of Developmental EtOH Exposure on Synaptic Plasticity

Developmental EtOH exposure can have a variety of detrimental effects on neuronal health, synapse development, synapse maturation and synaptic plasticity. While neuronal cell death could profoundly alter network function in FASD, there is ample evidence suggesting that the function of remaining neurons is altered. Because microglia have been intimately tied to the formation and removal of synapses during plasticity, it is important to review the deficits that have been observed in excitatory synaptic remodeling after developmental EtOH exposure.

The structure and function of dendritic spines, the postsynaptic structures of excitatory synapses, appear to be sensitive to developmental EtOH. An initial case study showed evidence that extreme amounts of fetal EtOH exposure could severely impair dendritic spine development, leading to reduced spine density and elongation of remaining spines (Ferrer and Galofre, 1987). This postmortem study involved a 4-month-old microcephalic child whose mother suffered from alcohol addiction and drank throughout pregnancy, and compared to age-matched controls. Similar findings have been reported in rat somatosensory cortex after exposure throughout rat gestation, although the effect was transient and resolved by adulthood (Galofre et al., 1987). EtOH exposure from E5 (embryonic day 5) to P0 (postnatal day 0, parturition) via dam intubation also showed that dendritic spine development on pyramidal neurons in visual cortex was delayed through adolescence in mice, with higher amounts of EtOH showing a more severe delay (Cui et al., 2010). Not all studies have described immature spine morphology, and the effects of EtOH on spines may be specific to the timing of EtOH exposure and brain area examined. For instance, decreases in spine density were found in rat prefrontal cortex after P4–P9 high binge EtOH exposure via intubation (Whitcher and Klintsova, 2008). In this case, no differences in either spine morphology or overall dendritic branching complexity were present.

Together, these findings suggest that developmental EtOH exposure can impair the formation of excitatory synapses, however, they do not address the dynamic nature of synapses, which are constantly remodeled as networks reorganize during plasticity. *In vivo* two-photon microscopy, which to our knowledge has not yet been applied to any FASD studies, has allowed synaptic structure and dynamics to be studied within the intact, living brain. This technique has shown that structure of synapses is constantly in flux, with dendritic spines not only changing their structural characteristics but also being lost and made *de novo*, both during development and, to a lesser degree, in adulthood (Grutzendler et al., 2002; Trachtenberg et al., 2002; Holtmaat et al., 2005; Zuo et al., 2005a). It has also been shown that smaller spines tend to represent weaker synapses that grow when the synapse is strengthened (Matsuzaki et al., 2004; Holtmaat et al., 2005), that learning or changes in experience can induce brain region specific spine formation and elimination (Zuo et al., 2005a,b; Majewska et al., 2006; Holtmaat et al., 2008), and that the ability of select newly formed spines to stabilize and become incorporated into the synaptic network long-term is essential for learning and memory (Xu et al., 2009; Yang et al., 2009). It will be interesting to determine whether the static deficits in spine development after developmental EtOH exposure also translate to deficits in spine remodeling in which spines are abnormally immature and therefore turn over at higher rates compared to age matched controls. Alternatively, it may be that developmental EtOH exposure causes an increased persistence of spines with immature morphology, with implications for altered synaptic function.

Indeed, functional measures of activity-dependent synaptic plasticity after developmental EtOH exposure are abnormal in multiple brain regions, including the hippocampus, cerebellum, prefrontal cortex and visual cortex. LTP in the hippocampus, a form of synaptic plasticity critical for learning and memory consolidation, is perhaps the most well studied form of synaptic plasticity studied in the context of prenatal alcohol exposure (as reviewed by Fontaine et al., 2016). Overall, the findings are variable, depending on EtOH exposure timing, dose, animal sex, age that plasticity was assessed, *in vivo* or *in vitro* electrophysiological measurement, differences in protocol to induce LTP, and whether the EtOH was administered to the whole animal prior to recordings or instead applied directly to the slice. Reductions in LTP within the CA1 region of hippocampus have been demonstrated after direct application of EtOH to P7–P9 rat hippocampal slice, as well as in slices from rats harvested hours after last exposure to EtOH vapor from P2 to P9 (Puglia and Valenzuela, 2010a,b). Both studies tested the possibility that the impaired LTP might result from reductions in glutamatergic synaptic strength and/or decreases in presynaptic glutamate release, but surprisingly found that EtOH exposure did not affect either of these parameters, but rather directly affected the increases in transmission elicited by stimulation protocols. In these studies, only the higher EtOH dose (approximately 300mg/dL) but not the lower EtOH dose (approximately 100mg/dL) impaired LTP, a general principal of dose dependence that has also been demonstrated by others (Bellinger et al., 2002; Puglia and Valenzuela, 2010a, b). In adolescent and adult animals, developmental EtOH exposure has been shown to reduce LTP in the CA1 (Richardson et al., 2002; Izumi et al., 2005; An et al., 2013), though several studies have also reported no effect on LTP (Bellinger et al., 1999; Krahl et al., 1999). Developmental EtOH exposure also reduces LTP in the dentate gyrus of adolescent and adult rodents (Sutherland et al., 1997; Sickmann et al., 2014), though this effect may be sex specific to males (Sickmann et al., 2014). A recent study focusing on EtOH’s effects on synaptogenesis on CA1 pyramidal cells in rat hippocampal organotypic slice cultures (direct application of EtOH to the slice for 7 days followed by maturation in culture without EtOH for 2–10 days) demonstrates that while control slice cultures over time showed increased expression of both pre and postsynaptic excitatory synapse proteins, had a high frequency of spontaneous excitatory postsynaptic currents (sEPSCs), and had organized microtubule ultrastructure at synapses, cultures with prior EtOH exposure had reduced pre and postsynaptic excitatory synaptic proteins, reduced sEPSC frequency, and grossly disorganized microtubule ultrastructure at synapses (Gerace et al., 2016). Overall, these results suggest that developmental EtOH exposure impairs activity-dependent synaptic plasticity in the hippocampus and is capable of causing delays in excitatory synapse development.

In the cerebellum, Purkinje cells that survive apoptotic cell death after EtOH exposure during the first and second trimesters exhibit LTP at the parallel fiber-Purkinje cell synapse with stimulation paradigms that normally induce LTD (Servais et al., 2007). Motor deficits (rotarod, runaway, conditioned eyelid blinking response) are also deficient in mice exposed to the same EtOH exposure paradigm, suggesting that the motor skill impairments in FASD are likely not a simple consequence of Purkinje cell loss, but also due to severe abnormalities in the network function of the Purkinje cells that remain (Servais et al., 2007). This may be a general phenomenon throughout the brain, where neurons previously exposed to EtOH alter their intrinsic and synaptic characteristics. A recent study focused on characterizing underlying causes of attention deficits in adult mice after a second and third trimester model of EtOH exposure. They report not only poorer performance on a behavioral task to assess visual attention, but also decreased intrinsic excitability of pyramidal neurons in layer VI of the medial prefrontal cortex, known to be important for normal attention (Louth et al., 2016). Furthermore, after BGS EtOH exposure, activity-dependent synaptic plasticity in visual cortex is also impaired (Medina et al., 2003; Medina and Ramoa, 2005; Lantz et al., 2012, 2015). In summary, it is clear that developmental EtOH exposure can have a long-term negative impact on a variety of mental processes that each rely on the plasticity of excitatory synapses in different brain regions. However, it remains unclear whether there may be a common mechanism underlying EtOH-induced delays in dendritic spine maturation as well as long-term defects in plasticity at excitatory synapses that translate into functional changes in cognition and behavior.

### Effects of EtOH-Induced Microglial Activation on Early Network Development

As discussed above, microglia carry out a number of important functions during early neuronal development which appear to be very sensitive to microglial state—release of trophic factors that regulate proliferation/survival vs. death in neurons and their progenitors and phagocytosis of apoptotic neurons (Figure 1). Even brief periods of inflammation may alter microglial actions and thus affect the trajectory of neural network development (Figure 2). During the BGS, microglia are in an immature state with amoeboid morphology and high expression of phagocytic markers (Shigemoto-Mogami et al., 2014), reminiscent of microglial phenotypes in anti-inflammatory conditions (Kettenmann et al., 2011). When microglial state is altered with agents such as minocycline, microglia are less efficient at carrying out their developmental functions (Shigemoto-Mogami et al., 2014; Miyamoto et al., 2016). This implies that EtOH exposure, which would temporarily shift the balance in the opposite direction, toward inflammation, could also interfere with microglial sculpting of neuronal populations, through apoptotic mechanisms that occur during early development. Such perturbations would change microglial expression profiles, affecting cytokine/chemokine production, growth factor release and phagocytic capabilities. This in turn could result in fewer neurons surviving, certain subpopulations of neurons being affected, increased cell death and insult to surviving neurons through the increase in extracellular debris that is not being phagocytosed. In fact, fractalkine signaling, which is critical for the survival of specific neurons (Ueno et al., 2013), has been explored in the context of developmental EtOH exposure. Fractalkine mRNA expression increases in the brains of wildtype mice 6 h after EtOH exposure on E8, but normalizes by E18 (Roberson et al., 2011), suggesting that this signaling pathway could modulate neuronal survival of certain populations of neurons after EtOH exposure. There is also evidence that the acute neuronal apoptotic response in mouse cortex within 6 h of EtOH exposure on P7 is exacerbated in both fractalkine and CX3CR1 null mice, as compared to wildtype mice (Sokolowski et al., 2014). At P7, CX3CR1 null microglia also exhibit increased association with apoptotic nuclei compared to CX3CR1 Het microglia (Sokolowski et al., 2014). Taken together, these findings suggest that EtOH exposure can have acute effects on fractalkine signaling with repercussions for neuronal survival and apoptosis, and that intact fractalkine signaling may serve as an early neuroprotective response to developmental EtOH exposure.

Early EtOH-induced microglial activation could also impact developing neural networks. Synaptogenesis is already occurring in some brain areas at these early times (Jiang and Nardelli, 2016) and changes in microglial cytokine and growth factor release, including changes to important synapse promoting molecules such as BDNF, could inhibit dendritic filopodia outgrowth and thus impede early synaptogenesis, as microglial state is critical to the outgrowth-promoting function of microglia (Miyamoto et al., 2016). In fact, neuroinflammation is known to affect the experience-dependent remodeling of neural connectivity throughout life (Smith et al., 2016) and may therefore interfere with the early establishment of networks during the time of EtOH exposure. Changes in complement signaling within microglia may inhibit microglial interactions with neurons that express C1q (Schafer et al., 2012), and an inflammatory brain milieu could alter TGF-β signaling within astrocytes (Bialas and Stevens, 2013), further impeding neuron-microglia signaling. EtOH-induced changes in fractalkine signaling (Roberson et al., 2011) could also alter microglial infiltration into regions were synaptic remodeling occurs (Paolicelli et al., 2011; Hoshiko et al., 2012; Arnoux et al., 2013), leading to the abnormal development of neural circuits, which could result in deficits in behavior (Zhan et al., 2014).

### Long-Term Changes in Microglial Behavior after Developmental EtOH Exposure

While PPAR-γ agonists hold promise for limiting EtOH-induced damage, it is unclear to what extent this treatment can alter long-term cognitive and behavioral effects in animals models (and eventually humans). One downside to this treatment avenue is that microglial activation appears to be limited to the timeframe of EtOH exposure and thus the treatment would need to be taken pre-emptively and concomitantly with EtOH exposure. A treatment effectively administered after FASD diagnosis would be most beneficial to all those currently living with FASD. From the research reviewed above, it is becoming increasingly clear that neurons and their networks do not develop normally even if those neurons escape death after EtOH exposure. Ideally a treatment would normalize these neuronal deficiencies and optimize network function in the previously exposed brain. It is unclear whether microglia can be such a target because thus far long-term deficits in microglial function due to developmental EtOH exposure have not been identified. This may be because current studies have primarily focused on how EtOH impacts the immune functions of microglia. As we learn more about how microglia carry out their roles in the normal brain, in the absence of immune activation, we may be able to use this knowledge to determine whether the physiological functions of microglia are altered after developmental EtOH exposure.

While there is little direct evidence of EtOH affecting non-immune activated microglial functions, there is some indirect evidence to suggest that targeting these functions may be a promising avenue to pursue. As discussed earlier, microglia in the healthy brain have finely branched and motile processes that physically contact dendritic spines, influencing both the proper formation and elimination of excitatory synapses throughout life and during experience-dependent synaptic plasticity (Nimmerjahn et al., 2005; Wake et al., 2009; Tremblay et al., 2010; Paolicelli et al., 2014; Miyamoto et al., 2016). While recent work has shown that microglial morphology appears to be intact long-term after developmental EtOH exposure (Topper et al., 2015), it remains unclear whether EtOH can elicit more subtle changes in microglial function that could persist into adulthood, including in the dynamic behavior of microglial processes, their physical interactions with synaptic elements or their ability to respond and provide cues to neurons. Deficits in plastic processes that have been directly tied to microglial function, such as ocular dominance plasticity (Sipe et al., 2016), exist in FASD models (Medina et al., 2003; Medina and Ramoa, 2005; Lantz et al., 2012, 2015), suggesting that EtOH-exposed microglia may contribute to these deficits. Additionally, the dendritic spine density and morphology deficits in FASD may be affected in part by inappropriate microglial signaling and pruning of synapses.

Given the important role of BDNF signaling in dendritic spine formation, focus on how developmental EtOH may affect BDNF production is warranted. Studies using *ex vivo* cultured fetal rat hypothalamic neurons have shown that these neurons reduce their production of BDNF in the presence of conditioned media taken from cultured microglia treated with EtOH (Boyadjieva and Sarkar, 2013), suggesting that EtOH could reduce general BDNF levels through microglia. On the other hand, enhanced spine removal by microglia could result from deregulated complement (Hong et al., 2016) or purinergic signaling (Abiega et al., 2016), two pathways which may act to recruit microglia to synapses in need of removal. Purinergic signaling in particular appears perfectly poised to guide microglial processes to synapses based on synaptic activity (Fontainhas et al., 2011; Li et al., 2012; Dissing-Olesen et al., 2014; Eyo et al., 2014). There are currently no studies that have explored whether developmental EtOH exposure alters these pathways long-term. There is evidence, however, that *in vitro* exposure of embryonic stem cell derived microglia to EtOH can upregulate the expression of P2RX4, a purinergic receptor, and may therefore affect microglial sensing of extracellular purines. This upregulation in turn severely impairs the chemotaxis of microglia toward fractalkine (Gofman et al., 2014), another pathway that has been implicated in microglial pruning of synapses. Therefore, EtOH-induced changes in P2RX4 expression on developing microglia could deregulate purinergic and fractalkine signaling, two different mechanisms of neuron-microglia crosstalk, and may therefore impact synaptic plasticity. Furthermore, a primary stimulus for the release of BDNF from microglia may be the binding of ATP to microglial P2RX4 (Parkhurst et al., 2013), suggesting that EtOH could also impact synaptic growth through this mechanism.

Directly profiling the transcriptome of microglia at different times after developmental EtOH exposure would yield more clues as to whether and how microglia behavior and responses to external cues change in developing and adult animals. While this has not been done, several studies have described changes in gene expression acutely and long after the end of developmental exposure in brain homogenates containing a mixed cell-type population (Chater-Diehl et al., 2016; Rogic et al., 2016; Pagé-Larivière et al., 2017). Many of these and other studies have identified developmental EtOH’s effects on genes that function in epigenetic regulation (Perkins et al., 2013; Ignacio et al., 2014; Subbanna et al., 2014; Rogic et al., 2016; Laufer et al., 2017). For example, in mouse hippocampus after even a single day (P7) of EtOH exposure at a level just above the 200 mg/dL toxic threshold, acute apoptosis is accompanied by increased levels of three proteins with activity that either causes histone methylation or demethylation (DNMT3a and G9a respectively) or binding to methylated sites to facilitate reduced gene transcription (MeCP2; Subbanna et al., 2014). mRNA transcript levels of MeCP2 and DMNT3a were also found to be altered in rat hippocampus after high binge EtOH exposure from GD1 through P10 (Perkins et al., 2013). This is particularly interesting in the context of the known modulation of BDNF expression by MeCP2 (Zhou et al., 2006). While the cell specificity of these epigenetic modifications caused by developmental EtOH exposure is not known, these studies suggest that changes in the epigenome could be affecting microglial function and deserve further attention. Much work is still needed to determine whether developmental EtOH exposure has lasting effects on how microglia and neurons communicate, and it is important that we understand these interactions even in a CNS environment that is not overtly neuroinflammatory.

## Conclusion

While recent work has begun to uncover the contributions of microglia to the circuit deficits elicited by developmental EtOH exposure, many important future research directions remain. It is becoming clear that immune activation could play a large role in how microglia affect the early development of the brain, but this immune activation resolves quickly and may be only part of the story of how microglia are affected by EtOH. To determine how long-term changes in microglial function could contribute to FASD symptoms, it will be important to understand the role of microglia in normal brain development and use the lessons learned to determine whether these non-immune functions of microglia change with developmental EtOH exposure.

## Author Contributions

All authors contributed to writing this manuscript.

## Conflict of Interest Statement

The authors declare that the research was conducted in the absence of any commercial or financial relationships that could be construed as a potential conflict of interest.

## References

[B1] Abiega O. Beccari S. Diaz-Aparicio I. Nadjar A. Laye S. Leyrolle Q.. (2016). Neuronal hyperactivity disturbs ATP microgradients, impairs microglial motility, and reduces phagocytic receptor expression triggering apoptosis/microglial phagocytosis uncoupling. PLoS Biol. 14:e1002466. 10.1371/journal.pbio.1002466 27228556 PMC4881984

[B2] Ahlers K. E. Karacay B. Fuller L. Bonthius D. J. Dailey M. E. (2015). Transient activation of microglia following acute alcohol exposure in developing mouse neocortex is primarily driven by BAX-dependent neurodegeneration. Glia 63, 1694–1713. 10.1002/glia.22835 25856413 PMC4534325

[B3] Ajami B. Bennett J. L. Krieger C. Tetzlaff W. Rossi F. M. (2007). Local self-renewal can sustain CNS microglia maintenance and function throughout adult life. Nat. Neurosci. 10, 1538–1543. 10.1038/nn2014 18026097

[B4] Alfonso-Loeches S. Pascual-Lucas M. Blanco A. M. Sanchez-Vera I. Guerri C. (2010). Pivotal role of TLR4 receptors in alcohol-induced neuroinflammation and brain damage. J. Neurosci. 30, 8285–8295. 10.1523/jneurosci.0976-10.2010 20554880 PMC6634595

[B5] Alliot F. Godin I. Pessac B. (1999). Microglia derive from progenitors, originating from the yolk sac and which proliferate in the brain. Brain Res. Dev. Brain Res. 117, 145–152. 10.1016/s0165-3806(99)00113-3 10567732

[B6] An L. Yang Z. Zhang T. (2013). Imbalanced synaptic plasticity induced spatial cognition impairment in male offspring rats treated with chronic prenatal ethanol exposure. Alcohol. Clin. Exp. Res. 37, 763–770. 10.1111/acer.12040 23240555

[B7] Arno B. Grassivaro F. Rossi C. Bergamaschi A. Castiglioni V. Furlan R.. (2014). Neural progenitor cells orchestrate microglia migration and positioning into the developing cortex. Nat. Commun. 5:5611. 10.1038/ncomms6611 25425146

[B8] Arnoux I. Hoshiko M. Mandavy L. Avignone E. Yamamoto N. Audinat E. (2013). Adaptive phenotype of microglial cells during the normal postnatal development of the somatosensory “Barrel” cortex. Glia 61, 1582–1594. 10.1002/glia.22503 23893820

[B9] Avalos L. A. Roberts S. C. Kaskutas L. A. Block G. Li D. K. (2014). Volume and type of alcohol during early pregnancy and the risk of miscarriage. Subst. Use Misuse 49, 1437–1445. 10.3109/10826084.2014.912228 24810392 PMC4183196

[B10] Bellinger F. P. Bedi K. S. Wilson P. Wilce P. A. (1999). Ethanol exposure during the third trimester equivalent results in long-lasting decreased synaptic efficacy but not plasticity in the CA1 region of the rat hippocampus. Synapse 31, 51–58. 10.1002/(SICI)1098-2396(199901)31:1<51::AID-SYN7>3.0.CO;2-O 10025683

[B11] Bellinger F. P. Davidson M. S. Bedi K. S. Wilce P. A. (2002). Neonatal ethanol exposure reduces AMPA but not NMDA receptor levels in the rat neocortex. Dev. Brain Res. 136, 77–84. 10.1016/s0165-3806(02)00363-2 12036520

[B12] Bialas A. R. Stevens B. (2013). TGF-β signaling regulates neuronal C1q expression and developmental synaptic refinement. Nat Neurosci 16, 1773–1782. 10.1038/nn.3560 24162655 PMC3973738

[B13] Block M. L. Zecca L. Hong J. S. (2007). Microglia-mediated neurotoxicity: uncovering the molecular mechanisms. Nat. Rev. Neurosci. 8, 57–69. 10.1038/nrn2038 17180163

[B14] Bodnar T. S. Hill L. A. Weinberg J. (2016). Evidence for an immune signature of prenatal alcohol exposure in female rats. Brain Behav. Immun. 58, 130–141. 10.1016/j.bbi.2016.05.022 27263429 PMC5067180

[B15] Boschen K. E. Ruggiero M. J. Klintsova A. Y. (2016). Neonatal binge alcohol exposure increases microglial activation in the developing rat hippocampus. Neuroscience 324, 355–366. 10.1016/j.neuroscience.2016.03.033 26996510 PMC4838517

[B16] Boyadjieva N. I. Sarkar D. K. (2013). Cyclic adenosine monophosphate and brain-derived neurotrophic factor decreased oxidative stress and apoptosis in developing hypothalamic neuronal cells: role of microglia. Alcohol. Clin. Exp. Res. 37, 1370–1379. 10.1111/acer.12104 23550806 PMC3706564

[B17] Butovsky O. Jedrychowski M. P. Moore C. S. Cialic R. Lanser A. J. Gabriely G.. (2014). Identification of a unique TGF-β—dependent molecular and functional signature in microglia. Nat. Neurosci. 17, 131–143. 10.1038/nn.3599 24316888 PMC4066672

[B18] Cardona A. E. Pioro E. P. Sasse M. E. Kostenko V. Cardona S. M. Dijkstra I. M.. (2006). Control of microglial neurotoxicity by the fractalkine receptor. Nat. Neurosci. 9, 917–924. 10.1038/nn1715 16732273

[B19] Carson M. J. Reilly C. R. Sutcliffe J. G. Lo D. (1998). Mature microglia resemble immature antigen-presenting cells. Glia 22, 72–85. 10.1002/(sici)1098-1136(199801)22:1<72::aid-glia7>3.0.co;2-a 9436789

[B20] Chater-Diehl E. J. Laufer B. I. Castellani C. A. Alberry B. L. Singh S. M. (2016). Alteration of gene expression, DNA methylation, and histone methylation in free radical scavenging networks in adult mouse hippocampus following fetal alcohol exposure. PLoS One 11:e0154836. 10.1371/journal.pone.0154836 27136348 PMC4852908

[B21] Clarren S. K. Alvord E. C. Jr. Sumi S. M. Streissguth A. P. Smith D. W. (1978). Brain malformations related to prenatal exposure to ethanol. J. Pediatr. 92, 64–67. 10.1016/s0022-3476(78)80072-9 619080

[B22] Corona A. W. Huang Y. O’Connor J. C. Dantzer R. Kelley K. W. Popovich P. G.. (2010). Fractalkine receptor (CX_3_CR1) deficiency sensitizes mice to the behavioral changes induced by lipopolysaccharide. J. Neuroinflammation 7:93. 10.1186/1742-2094-7-93 21167054 PMC3018416

[B23] Creeley C. E. Dikranian K. T. Johnson S. A. Farber N. B. Olney J. W. (2013). Alcohol-induced apoptosis of oligodendrocytes in the fetal macaque brain. Acta Neuropathol. Commun. 1:23. 10.1186/2051-5960-1-23 24252271 PMC3893424

[B24] Crews F. T. Qin L. Sheedy D. Vetreno R. P. Zou J. (2013). High mobility group box 1/Toll-like receptor danger signaling increases brain neuroimmune activation in alcohol dependence. Biol. Psychiatry 73, 602–612. 10.1016/j.biopsych.2012.09.030 23206318 PMC3602398

[B25] Cronk J. C. Derecki N. C. Ji E. Xu Y. Lampano A. E. Smirnov I.. (2015). Methyl-CpG binding protein 2 regulates microglia and macrophage gene expression in response to inflammatory stimuli. Immunity 42, 679–691. 10.1016/j.immuni.2015.03.013 25902482 PMC4407145

[B26] Cui Z. J. Zhao K. B. Zhao H. J. Yu D. M. Niu Y. L. Zhang J. S.. (2010). Prenatal alcohol exposure induces long-term changes in dendritic spines and synapses in the mouse visual cortex. Alcohol Alcohol. 45, 312–319. 10.1093/alcalc/agq036 20543181

[B27] Cunningham C. L. Martinez-Cerdeno V. Noctor S. C. (2013). Microglia regulate the number of neural precursor cells in the developing cerebral cortex. J. Neurosci. 33, 4216–4233. 10.1523/JNEUROSCI.3441-12.2013 23467340 PMC3711552

[B28] Davalos D. Grutzendler J. Yang G. Kim J. V. Zuo Y. Jung S.. (2005). ATP mediates rapid microglial response to local brain injury *in vivo*. Nat. Neurosci. 8, 752–758. 10.1038/nn1472 15895084

[B200] Del Río Hortega P. (1920). Estudios sobre la neuroglía. La microglía y su transformación en células en bastoncito y cuerpos granuloadiposos. Trab. Lab. Invest. Biol. 18, 37–82.

[B29] Derecki N. C. Cronk J. C. Lu Z. Xu E. Abbott S. B. Guyenet P. G.. (2012). Wild-type microglia arrest pathology in a mouse model of Rett syndrome. Nature 484, 105–109. 10.1038/nature10907 22425995 PMC3321067

[B30] Dissing-Olesen L. LeDue J. M. Rungta R. L. Hefendehl J. K. Choi H. B. MacVicar B. A. (2014). Activation of neuronal NMDA receptors triggers transient ATP-mediated microglial process outgrowth. J. Neurosci. 34, 10511–10527. 10.1523/jneurosci.0405-14.2014 25100586 PMC6802598

[B31] Dobbing J. Sands J. (1979). Comparative aspects of the brain growth spurt. Early Hum. Dev. 3, 79–83. 10.1016/0378-3782(79)90022-7 118862

[B32] Doney R. Lucas B. R. Watkins R. E. Tsang T. W. Sauer K. Howat P.. (2016). Visual-motor integration, visual perception, and fine motor coordination in a population of children with high levels of Fetal Alcohol Spectrum Disorder. Res. Dev. Disabil. 55, 346–357. 10.1016/j.ridd.2016.05.009 27228005

[B34] Drew P. D. Johnson J. W. Douglas J. C. Phelan K. D. Kane C. J. (2015). Pioglitazone blocks ethanol induction of microglial activation and immune responses in the hippocampus, cerebellum and cerebral cortex in a mouse model of fetal alcohol spectrum disorders. Alcohol. Clin. Exp. Res. 39, 445–454. 10.1111/acer.12639 25703036 PMC4348240

[B33] Drew P. D. Kane C. J. (2014). Fetal alcohol spectrum disorders and neuroimmune changes. Int. Rev. Neurobiol. 118, 41–80. 10.1016/b978-0-12-801284-0.00003-8 25175861 PMC4387770

[B35] Elmore M. R. Najafi A. R. Koike M. A. Dagher N. N. Spangenberg E. E. Rice R. A.. (2014). Colony-stimulating factor 1 receptor signaling is necessary for microglia viability, unmasking a microglia progenitor cell in the adult brain. Neuron 82, 380–397. 10.1016/j.neuron.2014.02.040 24742461 PMC4161285

[B36] Eyo U. B. Peng J. Y. Swiatkowski P. Mukherjee A. Bispo A. Wu L. J. (2014). Neuronal hyperactivity recruits microglial processes via neuronal NMDA receptors and microglial P2Y12 receptors after status epilepticus. J. Neurosci. 34, 10528–10540. 10.1523/jneurosci.0416-14.2014 25100587 PMC4200107

[B37] Feldman H. S. Jones K. L. Lindsay S. Slymen D. Klonoff-Cohen H. Kao K.. (2012). Prenatal alcohol exposure patterns and alcohol-related birth defects and growth deficiencies: a prospective study. Alcohol. Clin. Exp. Res. 36, 670–676. 10.1111/j.1530-0277.2011.01664.x 22250768

[B38] Fernandez-Lizarbe S. Pascual M. Guerri C. (2009). Critical role of TLR4 response in the activation of microglia induced by ethanol. J. Immunol. 183, 4733–4744. 10.4049/jimmunol.0803590 19752239

[B39] Ferrer I. Galofre E. (1987). Dendritic spine anomalies in fetal alcohol syndrome. Neuropediatrics 18, 161–163. 10.1055/s-2008-1052472 3683757

[B40] Fontaine C. J. Patten A. R. Sickmann H. M. Helfer J. L. Christie B. R. (2016). Effects of pre-natal alcohol exposure on hippocampal synaptic plasticity: sex, age and methodological considerations. Neurosci. Biobehav. Rev. 64, 12–34. 10.1016/j.neubiorev.2016.02.014 26906760

[B41] Fontainhas A. M. Wang M. Liang K. J. Chen S. Mettu P. Damani M.. (2011). Microglial morphology and dynamic behavior is regulated by ionotropic glutamatergic and GABAergic neurotransmission. PLoS One 6:e15973. 10.1371/journal.pone.0015973 21283568 PMC3026789

[B42] Frade J. M. Barde Y. A. (1998). Microglia-derived nerve growth factor causes cell death in the developing retina. Neuron 20, 35–41. 10.1016/s0896-6273(00)80432-8 9459440

[B43] Furtado E. F. Roriz S. T. (2016). Inattention and impulsivity associated with prenatal alcohol exposure in a prospective cohort study with 11-years-old Brazilian children. Eur. Child Adolesc. Psychiatry 25, 1327–1335. 10.1007/s00787-016-0857-y 27155839

[B44] Galofre E. Ferrer I. Fabregues I. Lopez-Tejero D. (1987). Effects of prenatal ethanol exposure on dendritic spines of layer V pyramidal neurons in the somatosensory cortex of the rat. J. Neurol. Sci. 81, 185–195. 10.1016/0022-510x(87)90095-5 3694227

[B45] Gerace E. Landucci E. Totti A. Bani D. Guasti D. Baronti R.. (2016). Ethanol toxicity during brain development: alterations of excitatory synaptic transmission in immature organotypic hippocampal slice cultures. Alcohol. Clin. Exp. Res. 40, 706–716. 10.1111/acer.13006 27038592

[B46] Ginhoux F. Greter M. Leboeuf M. Nandi S. See P. Gokhan S.. (2010). Fate mapping analysis reveals that adult microglia derive from primitive macrophages. Science 330, 841–845. 10.1126/science.1194637 20966214 PMC3719181

[B47] Giordano G. Guizzetti M. Dao K. Mattison H. A. Costa L. G. (2011). Ethanol impairs muscarinic receptor-induced neuritogenesis in rat hippocampal slices: role of astrocytes and extracellular matrix proteins. Biochem. Pharmacol. 82, 1792–1799. 10.1016/j.bcp.2011.08.014 21884684 PMC3205233

[B48] Gofman L. Cenna J. M. Potula R. (2014). P2X4 receptor regulates alcohol-induced responses in microglia. J. Neuroimmune Pharmacol. 9, 668–678. 10.1007/s11481-014-9559-8 25135400 PMC4209197

[B49] Goodlett C. R. Lundahl K. R. (1996). Temporal determinants of neonatal alcohol-induced cerebellar damage and motor performance deficits. Pharmacol. Biochem. Behav. 55, 531–540. 10.1016/s0091-3057(96)00248-1 8981583

[B50] Grabert K. Michoel T. Karavolos M. H. Clohisey S. Baillie J. K. Stevens M. P.. (2016). Microglial brain region-dependent diversity and selective regional sensitivities to aging. Nat. Neurosci. 19, 504–516. 10.1038/nn.4222 26780511 PMC4768346

[B51] Grutzendler J. Kasthuri N. Gan W. B. (2002). Long-term dendritic spine stability in the adult cortex. Nature 420, 812–816. 10.1038/nature01276 12490949

[B52] Guerri C. Pascual M. Renau-Piqueras J. (2001). Glia and fetal alcohol syndrome. Neurotoxicology 22, 593–599. 10.1016/s0161-813x(01)00037-7 11770880

[B53] Guizzetti M. Zhang X. Goeke C. Gavin D. P. (2014). Glia and neurodevelopment: focus on fetal alcohol spectrum disorders. Front. Pediatr. 2:123. 10.3389/fped.2014.00123 25426477 PMC4227495

[B54] Harrison J. K. Jiang Y. Chen S. Xia Y. Maciejewski D. McNamara R. K.. (1998). Role for neuronally derived fractalkine in mediating interactions between neurons and CX3CR1-expressing microglia. Proc. Natl. Acad. Sci. U S A 95, 10896–10901. 10.1073/pnas.95.18.10896 9724801 PMC27992

[B55] Haynes S. E. Hollopeter G. Yang G. Kurpius D. Dailey M. E. Gan W. B.. (2006). The P2Y_12_ receptor regulates microglial activation by extracellular nucleotides. Nat. Neurosci. 9, 1512–1519. 10.1038/nn1805 17115040

[B56] Hickman S. E. Kingery N. D. Ohsumi T. K. Borowsky M. L. Wang L. C. Means T. K.. (2013). The microglial sensome revealed by direct RNA sequencing. Nat. Neurosci. 16, 1896–1905. 10.1038/nn.3554 24162652 PMC3840123

[B57] Holtmaat A. De Paola V. Wilbrecht L. Knott G. W. (2008). Imaging of experience-dependent structural plasticity in the mouse neocortex *in vivo*. Behav. Brain Res. 192, 20–25. 10.1016/j.bbr.2008.04.005 18501438

[B58] Holtmaat A. J. Trachtenberg J. T. Wilbrecht L. Shepherd G. M. Zhang X. Knott G. W.. (2005). Transient and persistent dendritic spines in the neocortex *in vivo*. Neuron 45, 279–291. 10.1016/j.neuron.2005.01.003 15664179

[B60] Hong S. Beja-Glasser V. F. Nfonoyim B. M. Frouin A. Li S. Ramakrishnan S.. (2016). Complement and microglia mediate early synapse loss in Alzheimer mouse models. Science 352, 712–716. 10.1126/science.aad8373 27033548 PMC5094372

[B61] Hoshiko M. Arnoux I. Avignone E. Yamamoto N. Audinat E. (2012). Deficiency of the microglial receptor CX3CR1 impairs postnatal functional development of thalamocortical synapses in the barrel cortex. J. Neurosci. 32, 15106–15111. 10.1523/JNEUROSCI.1167-12.2012 23100431 PMC6704837

[B62] Idrus N. M. Napper R. M. (2012). Acute and long-term Purkinje cell loss following a single ethanol binge during the early third trimester equivalent in the rat. Alcohol. Clin. Exp. Res. 36, 1365–1373. 10.1111/j.1530-0277.2012.01743.x 22404759

[B63] Ignacio C. Mooney S. M. Middleton F. A. (2014). Effects of acute prenatal exposure to ethanol on microRNA expression are ameliorated by social enrichment. Front. Pediatr. 2:103. 10.3389/fped.2014.00103 25309888 PMC4173670

[B64] Ikonomidou C. Bittigau P. Ishimaru M. J. Wozniak D. F. Koch C. Genz K.. (2000). Ethanol-induced apoptotic neurodegeneration and fetal alcohol syndrome. Science 287, 1056–1060. 10.1126/science.287.5455.1056 10669420

[B65] Izumi Y. Nagashima K. Murayama K. Zorumski C. F. (2005). Acute effects of ethanol on hippocampal long-term potentiation and long-term depression are mediated by different mechanisms. Neuroscience 136, 509–517. 10.1016/j.neuroscience.2005.08.002 16216426

[B66] Ji K. Akgul G. Wollmuth L. P. Tsirka S. E. (2013). Microglia actively regulate the number of functional synapses. PLoS One 8:e56293. 10.1371/journal.pone.0056293 23393609 PMC3564799

[B67] Jiang X. Nardelli J. (2016). Cellular and molecular introduction to brain development. Neurobiol. Dis. 92, 3–17. 10.1016/j.nbd.2015.07.007 26184894 PMC4720585

[B68] Jung S. Aliberti J. Graemmel P. Sunshine M. J. Kreutzberg G. W. Sher A.. (2000). Analysis of fractalkine receptor CX_3_CR1 function by targeted deletion and green fluorescent protein reporter gene insertion. Mol. Cell. Biol. 20, 4106–4114. 10.1128/mcb.20.11.4106-4114.2000 10805752 PMC85780

[B69] Kalberg W. O. Provost B. Tollison S. J. Tabachnick B. G. Robinson L. K. Eugene Hoyme H.. (2006). Comparison of motor delays in young children with fetal alcohol syndrome to those with prenatal alcohol exposure and with no prenatal alcohol exposure. Alcohol. Clin. Exp. Res. 30, 2037–2045. 10.1111/j.1530-0277.2006.00250.x 17117969

[B70] Kane C. J. Drew P. D. (2016). Inflammatory responses to alcohol in the CNS: nuclear receptors as potential therapeutics for alcohol-induced neuropathologies. J. Leukoc. Biol. 100, 951–959. 10.1189/jlb.3mr0416-171r 27462100 PMC5069092

[B71] Kane C. J. Phelan K. D. Han L. Smith R. R. Xie J. Douglas J. C.. (2011). Protection of neurons and microglia against ethanol in a mouse model of fetal alcohol spectrum disorders by peroxisome proliferator-activated receptor-γ agonists. Brain Behav. Immun. 25, S137–S145. 10.1016/j.bbi.2011.02.016 21376806 PMC3104506

[B72] Kerns K. A. Siklos S. Baker L. Müller U. (2016). Emotion recognition in children with fetal alcohol spectrum disorders. Child Neuropsychol. 22, 255–275. 10.1080/09297049.2014.993310 25704232

[B73] Kettenmann H. Hanisch U. K. Noda M. Verkhratsky A. (2011). Physiology of microglia. Physiol. Rev. 91, 461–553. 10.1152/physrev.00011.2010 21527731

[B74] Kierdorf K. Erny D. Goldmann T. Sander V. Schulz C. Perdiguero E. G.. (2013). Microglia emerge from erythromyeloid precursors via Pu.1- and Irf8-dependent pathways. Nat. Neurosci. 16, 273–280. 10.1038/nn.3318 23334579

[B75] Krahl S. E. Berman R. F. Hannigan J. H. (1999). Electrophysiology of hippocampal CA1 neurons after prenatal ethanol exposure. Alcohol 17, 125–131. 10.1016/s0741-8329(98)00043-3 10064380

[B76] Lantz C. L. Sipe G. O. Wong E. L. Majewska A. K. Medina A. E. (2015). Effects of developmental alcohol exposure on potentiation and depression of visual cortex responses. Alcohol. Clin. Exp. Res. 39, 1434–1442. 10.1111/acer.12775 26108422 PMC4515209

[B77] Lantz C. L. Wang W. Medina A. E. (2012). Early alcohol exposure disrupts visual cortex plasticity in mice. Int. J. Dev. Neurosci. 30, 351–357. 10.1016/j.ijdevneu.2012.05.001 22617459 PMC3504720

[B78] Laufer B. I. Chater-Diehl E. J. Kapalanga J. Singh S. M. (2017). Long-term alterations to DNA methylation as a biomarker of prenatal alcohol exposure: from mouse models to human children with fetal alcohol spectrum disorders. Alcohol 60, 67–75. 10.1016/j.alcohol.2016.11.009 28187949

[B79] Lawrimore C. J. Crews F. T. (2017). Ethanol, TLR3, and TLR4 agonists have unique innate immune responses in neuron-like SH-SY5Y and microglia-like BV2. Alcohol. Clin. Exp. Res. 41, 939–954. 10.1111/acer.13368 28273337 PMC5407472

[B80] Lawson L. J. Perry V. H. Dri P. Gordon S. (1990). Heterogeneity in the distribution and morphology of microglia in the normal adult mouse brain. Neuroscience 39, 151–170. 10.1016/0306-4522(90)90229-w 2089275

[B81] Li Y. Du X. F. Liu C. S. Wen Z. L. Du J. L. (2012). Reciprocal regulation between resting microglial dynamics and neuronal activity *in vivo*. Dev. Cell 23, 1189–1202. 10.1016/j.devcel.2012.10.027 23201120

[B82] Liddelow S. A. Guttenplan K. A. Clarke L. E. Bennett F. C. Bohlen C. J. Schirmer L.. (2017). Neurotoxic reactive astrocytes are induced by activated microglia. Nature 541, 481–487. 10.1038/nature21029 28099414 PMC5404890

[B83] Light K. E. Belcher S. M. Pierce D. R. (2002). Time course and manner of Purkinje neuron death following a single ethanol exposure on postnatal day 4 in the developing rat. Neuroscience 114, 327–337. 10.1016/s0306-4522(02)00344-5 12204202

[B84] Louth E. L. Bignell W. Taylor C. L. Bailey C. D. (2016). Developmental ethanol exposure leads to long-term deficits in attention and its underlying prefrontal circuitry. eNeuro 3:ENEURO.0267-16.2016. 10.1523/eneuro.0267-16.2016 27844059 PMC5099605

[B85] Luo J. (2015). Effects of ethanol on the cerebellum: advances and prospects. Cerebellum 14, 383–385. 10.1007/s12311-015-0674-8 25933648 PMC4492805

[B86] Maggi L. Scianni M. Branchi I. D’Andrea I. Lauro C. Limatola C. (2011). CX_3_CR1 deficiency alters hippocampal-dependent plasticity phenomena blunting the effects of enriched environment. Front. Cell. Neurosci. 5:22. 10.3389/fncel.2011.00022 22025910 PMC3198035

[B87] Majewska A. K. Newton J. R. Sur M. (2006). Remodeling of synaptic structure in sensory cortical areas *in vivo*. J. Neurosci. 26, 3021–3029. 10.1523/JNEUROSCI.4454-05.2006 16540580 PMC6673961

[B88] Marín-Teva J. L. Dusart I. Colin C. Gervais A. van Rooijen N. Mallat M. (2004). Microglia promote the death of developing Purkinje cells. Neuron 41, 535–547. 10.1016/s0896-6273(04)00069-8 14980203

[B89] Matsuzaki M. Ellis-Davies G. C. Nemoto T. Miyashita Y. Iino M. Kasai H. (2001). Dendritic spine geometry is critical for AMPA receptor expression in hippocampal CA1 pyramidal neurons. Nat. Neurosci. 4, 1086–1092. 10.1038/nn736 11687814 PMC4229049

[B90] Matsuzaki M. Honkura N. Ellis-Davies G. C. Kasai H. (2004). Structural basis of long-term potentiation in single dendritic spines. Nature 429, 761–766. 10.1038/nature02617 15190253 PMC4158816

[B91] May P. A. Baete A. Russo J. Elliott A. J. Blankenship J. Kalberg W. O.. (2014). Prevalence and characteristics of fetal alcohol spectrum disorders. Pediatrics 134, 855–866. 10.1542/peds.2013-3319 25349310 PMC4210790

[B92] May P. A. de Vries M. M. Marais A. S. Kalberg W. O. Adnams C. M. Hasken J. M.. (2016). The continuum of fetal alcohol spectrum disorders in four rural communities in South Africa: prevalence and characteristics. Drug Alcohol Depend. 159, 207–218. 10.1016/j.drugalcdep.2015.12.023 26774945 PMC4724497

[B94] Medina A. E. Krahe T. E. Coppola D. M. Ramoa A. S. (2003). Neonatal alcohol exposure induces long-lasting impairment of visual cortical plasticity in ferrets. J. Neurosci. 23, 10002–10012. 14602814 10.1523/JNEUROSCI.23-31-10002.2003 PMC6740856

[B93] Medina A. E. Ramoa A. S. (2005). Early alcohol exposure impairs ocular dominance plasticity throughout the critical period. Dev. Brain Res. 157, 107–111. 10.1016/j.devbrainres.2005.03.012 15939092

[B95] Miller M. W. Potempa G. (1990). Numbers of neurons and glia in mature rat somatosensory cortex: effects of prenatal exposure to ethanol. J. Comp. Neurol. 293, 92–102. 10.1002/cne.902930108 2312794

[B96] Miller M. W. Robertson S. (1993). Prenatal exposure to ethanol alters the postnatal development and transformation of radial glia to astrocytes in the cortex. J. Comp. Neurol. 337, 253–266. 10.1002/cne.903370206 8276999

[B97] Miyamoto A. Wake H. Ishikawa A. W. Eto K. Shibata K. Murakoshi H.. (2016). Microglia contact induces synapse formation in developing somatosensory cortex. Nat. Commun. 7:12540. 10.1038/ncomms12540 27558646 PMC5007295

[B98] Nimmerjahn A. Kirchhoff F. Helmchen F. (2005). Resting microglial cells are highly dynamic surveillants of brain parenchyma *in vivo*. Science 308, 1314–1318. 10.1126/science.1110647 15831717

[B99] Nolte C. Möller T. Walter T. Kettenmann H. (1996). Complement 5a controls motility of murine microglial cells *in vitro* via activation of an inhibitory G-protein and the rearrangement of the actin cytoskeleton. Neuroscience 73, 1091–1107. 10.1016/0306-4522(96)00106-6 8809827

[B100] Olney J. W. Tenkova T. Dikranian K. Muglia L. J. Jermakowicz W. J. D’Sa C.. (2002). Ethanol-induced caspase-3 activation in the *in vivo* developing mouse brain. Neurobiol. Dis. 9, 205–219. 10.1006/nbdi.2001.0475 11895372

[B101] Pagé-Larivière F. Campagna C. Sirard M. A. (2017). Mechanisms involved in porcine early embryo survival following ethanol exposure. Toxicol. Sci. 156, 289–299. 10.1093/toxsci/kfw256 28069986

[B102] Paolicelli R. C. Bisht K. Tremblay M. E. (2014). Fractalkine regulation of microglial physiology and consequences on the brain and behavior. Front. Cell. Neurosci. 8:129. 10.3389/fncel.2014.00129 24860431 PMC4026677

[B103] Paolicelli R. C. Bolasco G. Pagani F. Maggi L. Scianni M. Panzanelli P.. (2011). Synaptic pruning by microglia is necessary for normal brain development. Science 333, 1456–1458. 10.1126/science.1202529 21778362

[B104] Parkhurst C. N. Yang G. Ninan I. Savas J. N. Yates J. R. III Lafaille J. J.. (2013). Microglia promote learning-dependent synapse formation through brain-derived neurotrophic factor. Cell 155, 1596–1609. 10.1016/j.cell.2013.11.030 24360280 PMC4033691

[B105] Paul A. P. Medina A. E. (2012). Overexpression of serum response factor in astrocytes improves neuronal plasticity in a model of early alcohol exposure. Neuroscience 221, 193–202. 10.1016/j.neuroscience.2012.06.045 22742904 PMC3504719

[B106] Peri F. Nüsslein-Volhard C. (2008). Live imaging of neuronal degradation by microglia reveals a role for v0-ATPase a1 in phagosomal fusion *in vivo*. Cell 133, 916–927. 10.1016/j.cell.2008.04.037 18510934

[B107] Perkins A. Lehmann C. Lawrence R. C. Kelly S. J. (2013). Alcohol exposure during development: impact on the epigenome. Int. J. Dev. Neurosci. 31, 391–397. 10.1016/j.ijdevneu.2013.03.010 23542005 PMC3703477

[B108] Perry V. H. Hume D. A. Gordon S. (1985). Immunohistochemical localization of macrophages and microglia in the adult and developing mouse brain. Neuroscience 15, 313–326. 10.1016/0306-4522(85)90215-5 3895031

[B109] Pfeiffer T. Avignone E. Nägerl U. V. (2016). Induction of hippocampal long-term potentiation increases the morphological dynamics of microglial processes and prolongs their contacts with dendritic spines. Sci. Rep. 6:32422. 10.1038/srep32422 27604518 PMC5015055

[B110] Pinazo-Duran M. D. Renau-Piqueras J. Guerri C. Strömland K. (1997). Optic nerve hypoplasia in fetal alcohol syndrome: an update. Eur. J. Ophthalmol. 7, 262–270. 9352281 10.1177/112067219700700311

[B111] Puglia M. P. Valenzuela C. F. (2010a). Ethanol acutely inhibits ionotropic glutamate receptor-mediated responses and long-term potentiation in the developing CA1 hippocampus. Alcohol. Clin. Exp. Res. 34, 594–606. 10.1111/j.1530-0277.2009.01128.x 20102565 PMC3050571

[B112] Puglia M. P. Valenzuela C. F. (2010b). Repeated third trimester-equivalent ethanol exposure inhibits long-term potentiation in the hippocampal CA1 region of neonatal rats. Alcohol 44, 283–290. 10.1016/j.alcohol.2010.03.001 20488644 PMC2916030

[B113] Qin L. Crews F. T. (2012). Chronic ethanol increases systemic TLR3 agonist-induced neuroinflammation and neurodegeneration. J. Neuroinflammation 9:130. 10.1186/1742-2094-9-130 22709825 PMC3412752

[B114] Qin L. He J. Hanes R. N. Pluzarev O. Hong J. S. Crews F. T. (2008). Increased systemic and brain cytokine production and neuroinflammation by endotoxin following ethanol treatment. J. Neuroinflammation 5:10. 10.1186/1742-2094-5-10 18348728 PMC2373291

[B115] Ransohoff R. M. El Khoury J. (2015). Microglia in health and disease. Cold Spring Harb. Perspect. Biol. 8:a020560. 10.1101/cshperspect.a020560 26354893 PMC4691795

[B116] Richardson D. P. Byrnes M. L. Brien J. F. Reynolds J. N. Dringenberg H. C. (2002). Impaired acquisition in the water maze and hippocampal long-term potentiation after chronic prenatal ethanol exposure in the guinea-pig. Eur. J. Neurosci. 16, 1593–1598. 10.1046/j.1460-9568.2002.02214.x 12405973

[B117] Roberson R. Kuddo T. Benassou I. Abebe D. Spong C. (2011). Neuroprotective fractalkine in fetal alcohol syndrome. Am. J. Obstet. Gynecol. 204, 400.e1–403.e3. 10.1016/j.ajog.2011.03.034 21572545 PMC3093089

[B118] Rogers J. T. Morganti J. M. Bachstetter A. D. Hudson C. E. Peters M. M. Grimmig B. A.. (2011). CX3CR1 deficiency leads to impairment of hippocampal cognitive function and synaptic plasticity. J. Neurosci. 31, 16241–16250. 10.1523/JNEUROSCI.3667-11.2011 22072675 PMC3236509

[B119] Rogic S. Wong A. Pavlidis P. (2016). Meta-analysis of gene expression patterns in animal models of prenatal alcohol exposure suggests role for protein synthesis inhibition and chromatin remodeling. Alcohol. Clin. Exp. Res. 40, 717–727. 10.1111/acer.13007 26996386 PMC5310543

[B120] Sasaki Y. Hoshi M. Akazawa C. Nakamura Y. Tsuzuki H. Inoue K.. (2003). Selective expression of Gi/o-coupled ATP receptor P2Y12 in microglia in rat brain. Glia 44, 242–250. 10.1002/glia.10293 14603465

[B121] Schafer D. P. Lehrman E. K. Kautzman A. G. Koyama R. Mardinly A. R. Yamasaki R.. (2012). Microglia sculpt postnatal neural circuits in an activity and complement-dependent manner. Neuron 74, 691–705. 10.1016/j.neuron.2012.03.026 22632727 PMC3528177

[B122] Sedel F. Béchade C. Vyas S. Triller A. (2004). Macrophage-derived tumor necrosis factor α, an early developmental signal for motoneuron death. J. Neurosci. 24, 2236–2246. 10.1523/JNEUROSCI.4464-03.2004 14999074 PMC6730439

[B123] Sekar A. Bialas A. R. de Rivera H. Davis A. Hammond T. R. Kamitaki N.. (2016). Schizophrenia risk from complex variation of complement component 4. Nature 530, 177–183. 10.1038/nature16549 26814963 PMC4752392

[B124] Servais L. Hourez R. Bearzatto B. Gall D. Schiffmann S. N. Cheron G. (2007). Purkinje cell dysfunction and alteration of long-term synaptic plasticity in fetal alcohol syndrome. Proc. Natl. Acad. Sci. U S A 104, 9858–9863. 10.1073/pnas.0607037104 17535929 PMC1887541

[B125] Shetty A. K. Phillips D. E. (1992). Effects of prenatal ethanol exposure on the development of Bergmann glia and astrocytes in the rat cerebellum: an immunohistochemical study. J. Comp. Neurol. 321, 19–32. 10.1002/cne.903210103 1613136

[B126] Shigemoto-Mogami Y. Hoshikawa K. Goldman J. E. Sekino Y. Sato K. (2014). Microglia enhance neurogenesis and oligodendrogenesis in the early postnatal subventricular zone. J. Neurosci. 34, 2231–2243. 10.1523/JNEUROSCI.1619-13.2014 24501362 PMC3913870

[B127] Sickmann H. M. Patten A. R. Morch K. Sawchuk S. Zhang C. Parton R.. (2014). Prenatal ethanol exposure has sex-specific effects on hippocampal long-term potentiation. Hippocampus 24, 54–64. 10.1002/hipo.22203 23996604

[B128] Sierra A. Abiega O. Shahraz A. Neumann H. (2013). Janus-faced microglia: beneficial and detrimental consequences of microglial phagocytosis. Front. Cell. Neurosci. 7:6. 10.3389/fncel.2013.00006 23386811 PMC3558702

[B129] Sierra A. Encinas J. M. Deudero J. J. Chancey J. H. Enikolopov G. Overstreet-Wadiche L. S.. (2010). Microglia shape adult hippocampal neurogenesis through apoptosis-coupled phagocytosis. Cell Stem Cell 7, 483–495. 10.1016/j.stem.2010.08.014 20887954 PMC4008496

[B130] Sipe G. O. Lowery R. L. Tremblay M.-E. Kelly E. A. Lamantia C. Majewska A. K. (2016). Microglial P2Y12 is necessary for synaptic plasticity in mouse visual cortex. Nat. Commun. 7:10905. 10.1038/ncomms10905 26948129 PMC4786684

[B131] Smith M. R. Burman P. Sadahiro M. Kidd B. A. Dudley J. T. Morishita H. (2016). Integrative analysis of disease signatures shows inflammation disrupts juvenile experience-dependent cortical plasticity. eNeuro 3:ENEURO.0240-16.2016. 10.1523/ENEURO.0240-16.2016 28101530 PMC5241709

[B132] Sokol R. J. Delaney-Black V. Nordstrom B. (2003). Fetal alcohol spectrum disorder. JAMA 290, 2996–2999. 10.1001/jama.290.22.2996 14665662

[B133] Sokolowski J. D. Chabanon-Hicks C. N. Han C. Z. Heffron D. S. Mandell J. W. (2014). Fractalkine is a “find-me” signal released by neurons undergoing ethanol-induced apoptosis. Front. Cell. Neurosci. 8:360. 10.3389/fncel.2014.00360 25426022 PMC4224129

[B134] Sood B. Delaney-Black V. Covington C. Nordstrom-Klee B. Ager J. Templin T.. (2001). Prenatal alcohol exposure and childhood behavior at age 6 to 7 years: I. dose-response effect. Pediatrics 108:E34. 10.1542/peds.108.2.e34 11483844

[B135] Sowell E. R. Johnson A. Kan E. Lu L. H. Van Horn J. D. Toga A. W.. (2008). Mapping white matter integrity and neurobehavioral correlates in children with fetal alcohol spectrum disorders. J. Neurosci. 28, 1313–1319. 10.1523/JNEUROSCI.5067-07.2008 18256251 PMC3567846

[B136] Squarzoni P. Oller G. Hoeffel G. Pont-Lezica L. Rostaing P. Low D.. (2014). Microglia modulate wiring of the embryonic forebrain. Cell Rep. 8, 1271–1279. 10.1016/j.celrep.2014.07.042 25159150

[B137] Stephan A. H. Barres B. A. Stevens B. (2012). The complement system: an unexpected role in synaptic pruning during development and disease. Annu. Rev. Neurosci. 35, 369–389. 10.1146/annurev-neuro-061010-113810 22715882

[B138] Stephen J. M. Kodituwakku P. W. Kodituwakku E. L. Romero L. Peters A. M. Sharadamma N. M.. (2012). Delays in auditory processing identified in preschool children with FASD. Alcohol. Clin. Exp. Res. 36, 1720–1727. 10.1111/j.1530-0277.2012.01769.x 22458372 PMC3390452

[B139] Stevens B. Allen N. J. Vazquez L. E. Howell G. R. Christopherson K. S. Nouri N.. (2007). The classical complement cascade mediates CNS synapse elimination. Cell 131, 1164–1178. 10.1016/j.cell.2007.10.036 18083105

[B140] Stevens S. A. Major D. Rovet J. Koren G. Fantus E. Nulman I.. (2012). Social problem solving in children with fetal alcohol spectrum disorders. J. Popul. Ther. Clin. Pharmacol. 19, e99–e110. Available online at: http://www.jptcp.com/abstract/social-problem-solving-in-children-with-fetal-alcohol-spectrum-disorders-37370.html 22535836

[B141] Streissguth A. P. Barr H. M. Olson H. C. Sampson P. D. Bookstein F. L. Burgess D. M. (1994a). Drinking during pregnancy decreases word attack and arithmetic scores on standardized tests: adolescent data from a population-based prospective study. Alcohol. Clin. Exp. Res. 18, 248–254. 10.1111/j.1530-0277.1994.tb00009.x 8048722

[B143] Streissguth A. P. Sampson P. D. Olson H. C. Bookstein F. L. Barr H. M. Scott M.. (1994b). Maternal drinking during pregnancy: attention and short-term memory in 14-year-old offspring–a longitudinal prospective study. Alcohol. Clin. Exp. Res. 18, 202–218. 10.1111/j.1530-0277.1994.tb00904.x 8198221

[B142] Streissguth A. P. Bookstein F. L. Barr H. M. Sampson P. D. O’Malley K. Young J. K. (2004). Risk factors for adverse life outcomes in fetal alcohol syndrome and fetal alcohol effects. J. Dev. Behav. Pediatr. 25, 228–238. 10.1097/00004703-200408000-00002 15308923

[B144] Subbanna S. Nagre N. N. Shivakumar M. Umapathy N. S. Psychoyos D. Basavarajappa B. S. (2014). Ethanol induced acetylation of histone at G9a exon1 and G9a-mediated histone H3 dimethylation leads to neurodegeneration in neonatal mice. Neuroscience 258, 422–432. 10.1016/j.neuroscience.2013.11.043 24300108 PMC3954640

[B145] Sutherland R. J. McDonald R. J. Savage D. D. (1997). Prenatal exposure to moderate levels of ethanol can have long-lasting effects on hippocampal synaptic plasticity in adult offspring. Hippocampus 7, 232–238. 10.1002/(SICI)1098-1063(1997)7:2<232::AID-HIPO9>3.0.CO;2-O 9136052

[B146] Swinnen N. Smolders S. Avila A. Notelaers K. Paesen R. Ameloot M.. (2013). Complex invasion pattern of the cerebral cortex bymicroglial cells during development of the mouse embryo. Glia 61, 150–163. 10.1002/glia.22421 23001583

[B147] Tan C. H. Denny C. H. Cheal N. E. Sniezek J. E. Kanny D. (2015). Alcohol use and binge drinking among women of childbearing age–United States, 2011–2013. MMWR Morb. Mortal. Wkly. Rep. 64, 1042–1046. 10.15585/mmwr.mm6437a3 26401713

[B148] Tenkova T. Young C. Dikranian K. Labruyere J. Olney J. W. (2003). Ethanol-induced apoptosis in the developing visual system during synaptogenesis. Invest. Ophthalmol. Vis. Sci. 44, 2809–2817. 10.1167/iovs.02-0982 12824217

[B149] Tomás M. Marín P. Megías L. Egea G. Renau-Piqueras J. (2005). Ethanol perturbs the secretory pathway in astrocytes. Neurobiol. Dis. 20, 773–784. 10.1016/j.nbd.2005.05.012 15953732

[B150] Topper L. A. Baculis B. C. Valenzuela C. F. (2015). Exposure of neonatal rats to alcohol has differential effects on neuroinflammation and neuronal survival in the cerebellum and hippocampus. J. Neuroinflammation 12:160. 10.1186/s12974-015-0382-9 26337952 PMC4558631

[B151] Torres L. Danver J. Ji K. Miyauchi J. T. Chen D. Anderson M. E.. (2016). Dynamic microglial modulation of spatial learning and social behavior. Brain Behav. Immun. 55, 6–16. 10.1016/j.bbi.2015.09.001 26348580 PMC4779430

[B152] Trachtenberg J. T. Chen B. E. Knott G. W. Feng G. Sanes J. R. Welker E.. (2002). Long-term *in vivo* imaging of experience-dependent synaptic plasticity in adult cortex. Nature 420, 788–794. 10.1038/nature01273 12490942

[B153] Tremblay M. E. Lowery R. L. Majewska A. K. (2010). Microglial interactions with synapses are modulated by visual experience. PLoS Biol. 8:e1000527. 10.1371/journal.pbio.1000527 21072242 PMC2970556

[B154] Tropea D. Majewska A. K. Garcia R. Sur M. (2010). Structural dynamics of synapses *in vivo* correlate with functional changes during experience-dependent plasticity in visual cortex. J. Neurosci. 30, 11086–11095. 10.1523/JNEUROSCI.1661-10.2010 20720116 PMC2932955

[B155] Ueno M. Fujita Y. Tanaka T. Nakamura Y. Kikuta J. Ishii M.. (2013). Layer V cortical neurons require microglial support for survival during postnatal development. Nat. Neurosci. 16, 543–551. 10.1038/nn.3358 23525041

[B156] Vallés S. Sancho-Tello M. Minana R. Climent E. Renau-Piqueras J. Guerri C. (1996). Glial fibrillary acidic protein expression in rat brain and in radial glia culture is delayed by prenatal ethanol exposure. J. Neurochem. 67, 2425–2433. 10.1046/j.1471-4159.1996.67062425.x 8931475

[B157] Vela J. M. Dalmau I. Gonzalez B. Castellano B. (1995). Morphology and distribution of microglial cells in the young and adult mouse cerebellum. J. Comp. Neurol. 361, 602–616. 10.1002/cne.903610405 8576417

[B158] Vernescu R. M. Adams R. J. Courage M. L. (2012). Children with fetal alcohol spectrum disorder show an amblyopia-like pattern of vision deficit. Dev. Med. Child Neurol. 54, 557–562. 10.1111/j.1469-8749.2012.04254.x 22574626

[B159] Wake H. Moorhouse A. J. Jinno S. Kohsaka S. Nabekura J. (2009). Resting microglia directly monitor the functional state of synapses *in vivo* and determine the fate of ischemic terminals. J. Neurosci. 29, 3974–3980. 10.1523/JNEUROSCI.4363-08.2009 19339593 PMC6665392

[B160] Wakselman S. Bechade C. Roumier A. Bernard D. Triller A. Bessis A. (2008). Developmental neuronal death in hippocampus requires the microglial CD11b integrin and DAP12 immunoreceptor. J. Neurosci. 28, 8138–8143. 10.1523/JNEUROSCI.1006-08.2008 18685038 PMC6670768

[B161] Whitcher L. T. Klintsova A. Y. (2008). Postnatal binge-like alcohol exposure reduces spine density without affecting dendritic morphology in rat mPFC. Synapse 62, 566–573. 10.1002/syn.20532 18512209 PMC10156950

[B162] Wozniak D. F. Hartman R. E. Boyle M. P. Vogt S. K. Brooks A. R. Tenkova T.. (2004). Apoptotic neurodegeneration induced by ethanol in neonatal mice is associated with profound learning/memory deficits in juveniles followed by progressive functional recovery in adults. Neurobiol. Dis. 17, 403–414. 10.1016/j.nbd.2004.08.006 15571976

[B163] Xu T. Yu X. Perlik A. J. Tobin W. F. Zweig J. A. Tennant K.. (2009). Rapid formation and selective stabilization of synapses for enduring motor memories. Nature 462, 915–919. 10.1038/nature08389 19946267 PMC2844762

[B164] Yang G. Pan F. Gan W. B. (2009). Stably maintained dendritic spines are associated with lifelong memories. Nature 462, 920–924. 10.1038/nature08577 19946265 PMC4724802

[B165] Yuan J. Lipinski M. Degterev A. (2003). Diversity in the mechanisms of neuronal cell death. Neuron 40, 401–413. 10.1016/s0896-6273(03)00601-9 14556717

[B166] Zhan Y. Paolicelli R. C. Sforazzini F. Weinhard L. Bolasco G. Pagani F.. (2014). Deficient neuron-microglia signaling results in impaired functional brain connectivity and social behavior. Nat. Neurosci. 17, 400–406. 10.1038/nn.3641 24487234

[B167] Zhang Y. Chen K. Sloan S. A. Bennett M. L. Scholze A. R. O’Keeffe S.. (2014). An RNA-sequencing transcriptome and splicing database of glia, neurons, and vascular cells of the cerebral cortex. J. Neurosci. 34, 11929–11947. 10.1523/JNEUROSCI.1860-14.2014 25186741 PMC4152602

[B168] Zhou Q. Homma K. J. Poo M. M. (2004). Shrinkage of dendritic spines associated with long-term depression of hippocampal synapses. Neuron 44, 749–757. 10.1016/j.neuron.2004.11.011 15572107

[B169] Zhou Z. Hong E. J. Cohen S. Zhao W. N. Ho H. Y. Schmidt L.. (2006). Brain-specific phosphorylation of MeCP2 regulates activity-dependent Bdnf transcription, dendritic growth and spine maturation. Neuron 52, 255–269. 10.1016/j.neuron.2006.09.037 17046689 PMC3962021

[B170] Zuo Y. Lin A. Chang P. Gan W. B. (2005a). Development of long-term dendritic spine stability in diverse regions of cerebral cortex. Neuron 46, 181–189. 10.1016/j.neuron.2005.04.001 15848798

[B172] Zuo Y. Yang G. Kwon E. Gan W. B. (2005b). Long-term sensory deprivation prevents dendritic spine loss in primary somatosensory cortex. Nature 436, 261–265. 10.1038/nature03715 16015331

